# Covalent Triazine Frameworks Based on the First *Pseudo*-Octahedral Hexanitrile Monomer via Nitrile Trimerization: Synthesis, Porosity, and CO_2_ Gas Sorption Properties

**DOI:** 10.3390/ma14123214

**Published:** 2021-06-10

**Authors:** Isabelle D. Wessely, Alexandra M. Schade, Subarna Dey, Asamanjoy Bhunia, Alexander Nuhnen, Christoph Janiak, Stefan Bräse

**Affiliations:** 1 Institute of Organic Chemistry (IOC), Karlsruhe Institute of Technology (KIT), Fritz-Haber-Weg 6, D-76131 Karlsruhe, Germany; isabelle.wessely@t-online.de (I.D.W.); alexandra-schade@gmx.de (A.M.S.); 2 Herbstreith & Fox GmbH & Co. KG Pektin-Fabriken, D-75305 Neuenbürg, Germany; 3 Institute of Inorganic and Structural Chemistry, Heinrich-Heine-University Düsseldorf, D-40204 Düsseldorf, Germany; subarna.chemie@gmail.com (S.D.); Alexander.Nuhnen@uni-duesseldorf.de (A.N.); janiak@uni-duesseldorf.de (C.J.); 4 Department of Chemistry, Inorganic Chemistry Section, Jadavpur University, Jadavpur, Kolkata 700032, India; asamanjoy.bhunia@gmail.com; 5 Institute of Biological and Chemical Systems (IBCS-FMS), Karlsruhe Institute of Technology (KIT), Hermann-von-Helmholtz-Platz 1, D-76344 Eggenstein-Leopoldshafen, Germany

**Keywords:** covalent triazine frameworks, CTFs, carbon dioxide adsorption, pseudo-octahedral hexanitrile, mixed linker

## Abstract

Herein, we report the first synthesis of covalent triazine-based frameworks (CTFs) based on a hexanitrile monomer, namely the novel pseudo-octahedral hexanitrile 1,4-bis(tris(4′-cyano-phenyl)methyl)benzene **1** using both ionothermal reaction conditions with ZnCl_2_ at 400 °C and the milder reaction conditions with the strong Brønsted acid trifluoromethanesulfonic acid (TFMS) at room temperature. Additionally, the hexanitrile was combined with different di-, tri-, and tetranitriles as a second linker based on recent work of mixed-linker CTFs, which showed enhanced carbon dioxide captures. The obtained framework structures were characterized via infrared (IR) spectroscopy, elemental analysis, scanning electron microscopy (SEM), and gas sorption measurements. Nitrogen adsorption measurements were performed at 77 K to determine the Brunauer-Emmett-Teller (BET) surface areas range from 493 m^2^/g to 1728 m^2^/g (*p*/*p*_0_ = 0.01–0.05). As expected, the framework **CTF-hex6** synthesized from **1** with ZnCl_2_ possesses the highest surface area for nitrogen adsorption. On the other hand, the mixed framework structure **CTF-hex4** formed from the hexanitrile **1** and 1,3,5 tricyanobenzene (**4**) shows the highest uptake of carbon dioxide and methane of 76.4 cm^3^/g and 26.6 cm^3^/g, respectively, at 273 K.

## 1. Introduction

Porous solids such as metal-organic frameworks (MOFs) [1,2,3,4], covalent organic frameworks (COFs) [5,6,7,8,9], porous organic polymers (POPs), and microporous organic polymers (MOPs) with adsorption properties due to a high surface area are widely used for gas separation and storage [10,11,12,13]. Especially, porous organic polymers are excellent candidates because of their high thermal and chemical stability, wide synthetic diversity as well as stability against water and acidic conditions [5,14]. A range of MOPs and POPs, which are often differentiated according to their tectons, have been developed, such as, hyper-crosslinked polymers (HCPs) [15,16], polymers of intrinsic microporosity (PIMs) [17,18], porous aromatic frameworks (PAFs) [14,19], conjugated microporous polymers (CMPs) [20,21], porous polymer networks (PPNs) [22,23] or porous covalent triazine-based frameworks (CTFs) [24,25,26,27,28]. Since their first synthesis by Kuhn et al. in 2008 [24,29,30], CTFs have received considerable attention for CO_2_ adsorption [27,31,32,33,34,35,36,37,38,39,40,41]. Post-combustion capture of CO_2_ is of great interest since CO_2_ is one of the main components influencing global warming [42,43]. To improve the CO_2_ uptake in porous polymers, π-systems and nitrogen atoms have been incorporated to achieve strong electrostatic interactions between the quadrupole moment of CO_2_ molecules and the heteroatoms or π-clouds of the pore walls also at low pressures [26,31,44].

Kuhn et al. developed an ionothermal synthesis method for trimerizing aromatic nitriles to triazine-based framework structures with permanent porosity and high thermal and chemical stabilities using anhydrous ZnCl_2_ at high temperatures [24,29,30]. Molten ZnCl_2_ acts as a solvent for the aromatic nitriles, as a Lewis acid catalyst, and as a pore-forming solvent and, therefore, as a templating agent for the polymerization [24,26,45,46]. Reaction temperatures of around 400 °C lead to lower BET surface areas (<2000 m^2^/g) than reaction temperatures of around 600 °C (>3000 m^2^/g possible) [29]. However, decomposition and condensation reactions such as C–H bond cleavage and carbonization occur, leading to a nitrogen deficiency in the elemental composition compared to their idealized structure [29,47].

Cooper et al. developed a method using the strong Brønsted acid trifluoromethanesulfonic acid (TFMS) at room temperature or under microwave conditions to avoid these decomposition reactions [25]. Besides the mild reaction conditions, the CTF synthesis with TFMS exhibits more advantages, such as short reaction times and the absence of ZnCl_2_ contaminations [48]. In contrast to CTFs formed via ionothermal conditions, the TFMS method provides lower surface areas and reduced nitrogen adsorption [25,29]. CTFs can also be synthesized by Friedel–Crafts reaction, e.g., from cyanuric chloride and aromatic hydrocarbons in the presence of AlCl_3_ [48,49,50,51,52,53,54,55]. Further, mechanochemical synthesis is a solvent-free alternative for CTF synthesis using the Friedel-Crafts route [56]. Highly crystalline CTFs were obtained due to the control of the nucleation by in situ formation of aldehyde monomers through the controlled oxidation of alcohols. The aromatic dialdehyde is then reacted with terephthalimidamide in a polycondensation reaction in DMSO in the presence of Cs_2_CO_3_ under air to form the triazine units. The BET surface areas of the CTFs from this synthetic approach were, however, relatively low (<600 m^2^/g) [57,58]. Higher surface areas with crystalline CTFs were reported from the condensation of aromatic diamides with P_4_O_10_ at 200 °C [59].

To conclude, the preliminary works of Kuhn et al. [24,29,30] and Cooper et al. [25,57] prompted us to investigate these strategies to novel core structures such as the HPX systems introduced by us [60].

## 2. Materials and Methods

### 2.1. General Remarks

Solvents, reagents, and chemicals were purchased from Sigma-Aldrich, ABCR, Acros Organics, and Fisher Scientific. All solvents, reagents, and chemicals were used as purchased unless stated otherwise. Absolute solvents were purchased from commercial suppliers (abs. DMF (Acros Organics, Fair Lawn, NJ, USA, <50 ppm water), absolute chloroform (Fischer Scientific GmbH, Nidderau, Germany, extra dry over molecular sieves), abs. NMP (N-methyl-2-pyrrolidinone, Fischer Scientific GmbH, Nidderau, Germany, <50 ppm water)). Reactions with air- or water-sensitive reagents were performed under Argon using standard Schlenk techniques. The synthesis of triazine frameworks under ionothermal conditions was performed in a tube furnace LOBA-1200-50-400-1-OW from HTM Reetz GmbH. The syntheses of 1,4-bis(tris(4′-cyanophenyl)methyl)benzene (**1**), 4,4′-dicyano-1,1′-biphenyl (**3**), 1,3,5-tricyanobenzene (**4**), and tetrakis(4-cyanophenyl)methane (**5**) are given in the Supplementary Information.

### 2.2. Gas Adsorption

Nitrogen sorption isotherms for **CTF-hex2** to **CTF-hex5** at 77 K were obtained using a NOVA-4000e instrument and a Thermo Scientific gas-adsorption-porosimeter for **CTF-hex1**. DFT calculations for the pore size distribution curves were done with the native ASWin 2.03 software from Quantachrome Instruments using the ‘N_2_ at 77 K on carbon, slit pore, nonlinear density functional theory (NLDFT) equilibrium’ model as well as the ‘N_2_ at 77 K on carbon, slit pore, quenched solid density functional theory (QSDFT) adsorption branch and equilibrium’ model, which is favorable for disordered micro/mesoporous carbon materials. CO_2_ and CH_4_ (and N_2_ for **CTF-hex6**) sorption isotherms were measured with a Micromeritics ASAP 2020 automatic gas sorption analyzer. The instrument is equipped with oil-free vacuum pumps, which deliver an ultimate vacuum of less than 10^−8^ mbar) and valves to allow contamination-free measurements. All gases (H_2_, He, N_2_, CO_2_, and CH_4_) were of ultrahigh purity (UHP, grade 5.0, 99.999%), and the standard temperature and pressure (STP) gas uptake volumes are reported in line with the NIST standards, which are at 293.15 K and 101.325 kPa. N_2_ sorption isotherms were recorded at 77 K (liquid nitrogen cooling). CO_2_ and CH_4_ sorption isotherms were measured at 293 ± 1 K and 273.15 K with the temperature set by a passive thermostat and an ice/deionized water bath, respectively. The density functional theory (DFT) pore size distributions from CO_2_ were based on the ‘NLDFT slit pore’ model using the ASAP 2020 v3.05 software.

### 2.3. Synthesis of **CTF-hex1**–**6**

General procedure with trifluoromethanesulfonic acid:

Under an argon atmosphere in a closed 20 mL vial, trifluoromethanesulfonic acid and chloroform (3.0 mL) were cooled to 0 °C. At this temperature, BTB-nitrile **1** (1.00 eq) and the respective aryl nitrile linker (3.00 eq for di-, 2.00 eq for tri-, and 0.60 eq for tetratopic tectones) dissolved in 10 mL chloroform were added over 30 min. The mixture was stirred for another 2 h at 0 °C and afterward at room temperature overnight. Then, the reaction mixture was poured on a water/NH_3(aq)_-mixture (100 mL, 20:1) and stirred at room temperature for an additional 2 h. The precipitate was filtered off, washed with distilled water (3 × 10 mL), ethanol (3 × 10 mL), acetone (3 × 10 mL), and chloroform (3 × 10 mL), and dried under high vacuum at 120 °C for 2 d to yield pale, light-yellow powders. For further details and analytical data, see in the Supplementary Information.

#### Ionothermal Synthesis

A mixture of 88.0 mg (123 μmol, 1.00 eq.) BTB-nitrile **1** and 168 mg (1.23 mmol, 10.0 eq.) dry ZnCl_2_ were heated in an oven up to 400 °C in a Pyrex^®^ ampule (3 mm × 120 mm) for 42 h. After cooling to room temperature, the ampule was opened carefully. The solid residue was washed with water (200 mL), stirred in dilute HCl (15 mL) overnight, and filtered as well as washed with water (3 × 10 mL) and tetrahydrofuran (3 × 10 mL). The obtained black solid was dried under a high vacuum (150 °C and 10^−6^ mbar); for analytical data, see in the Supplementary Information.

## 3. Results

In previous work, we investigated the synthesis of CTFs with various linker systems such as, for example, di-, tri-, and tetra-substituted adamantane derivatives [61] or tetra(4-cyanophenyl)ethylene [45,61]. In the latter case, ionothermal [62] and strong Brønsted [29] reaction conditions were used, respectively, for the framework synthesis. In dependence on earlier literature results, the nitrogen BET surface areas for the frameworks synthesized with TFMS were much lower. On the other hand, the CO_2_ and CH_4_ uptakes are in similar ranges [45,63]. While a mixed-linker assembly strategy is already widely applied to metal-ligand coordination polymers [64,65] and is also known, for example, for imine-based COFs [62], to the best of our knowledge, mixed-linker CTFs were only recently reported [66]. Recently, we could show that combining two nitrile linkers positively influences the framework structures and properties [46,66].

**Scheme 1:**
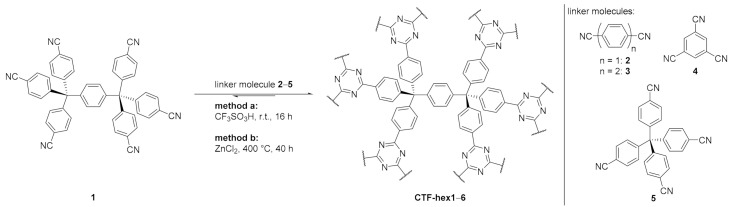
Synthesized triazine-based frameworks **CTF-hex1**–**6** with monomers **1**–**5** via nitrile trimerization with the strong Brønsted acid trifluoromethanesulfonic acid (**method a**) or via ionothermal reaction conditions (**method b**). The latter was only done with monomer **1**.

The use of tetrahedral adamantane derivatives as well as the successful mixed-building block approach motivated us to transfer this approach on another multi-nitrile linker structure, the *pseudo*-octahedral 1,4-bis(tris(4′-cyanophenyl)methyl)benzene (BTB-nitrile, **1**), which is, to the best of our knowledge, the first hexanitrile used in CTF preparation [60]. We combined this hexanitrile **1** with different planar dinitriles **2** and **3** and a trinitrile 4 and a tetrahedral tetraphenylmethane base nitrile **5** (reaction Scheme 1, Table 1).

### 3.1. Synthesis of Covalent Triazine Frameworks **CTF-hex1**–**6**

As described before, ionothermal reaction conditions are optimal for synthesizing triazine-based covalent organic frameworks with very high surface areas [13,24,25,26,27,28]; we used the novel *pseudo*-octahedral hexanitrile 1,4-bis(tris(4′-cyanophenyl)methyl)benzene **1** as tectone and investigated the framework formation with dry ZnCl_2_ under vacuum at 400 °C. According to previous work [46,63], a molar ratio of monomer to ZnCl_2_ of 1:10 leads to a higher surface area [14]. Therefore, this ratio was also used in the present work. A black solid in moderate to good yield was obtained (Table 1, entry 6). 

Because of the instability of some organic molecules under ionothermal reaction conditions, the milder conditions from Cooper et al. [25] with TFMS at room temperature were used to synthesize triazine-based frameworks with two building blocks in which *pseudo*-octahedral hexanitrile **1** was always used as a tectone (Table 1, entries 2–5). The two different linkers were used in an equimolar ratio for the nitrile moieties. To better compare with the triazine-based framework **CTF-hex6**, the hexanitrile **1** was first reacted with itself using TFMS (Table 1, entry 1). All triazine frameworks **CTF-hex1**–**5** synthesized with TFMS were isolated as slightly yellow powders.

The produced framework structures **CTF-hex1**–**6** were characterized via IR spectroscopy, elemental analysis, scanning electron microscopy (SEM), and gas sorption measurements. The elemental analyses show deviations from the calculated values for a hypothetical full-conversion (Table S2, Supplementary Information). Such deviations are reported in the literature due to incomplete conversion, adsorption of water or other molecules, and decomposition during the reaction [25,26,46,47,66,67]. The decreased amount of nitrogen, e.g., for **CTF-hex1** calculated 11.79%, found 9.27%, indicates the elimination of nitrogen species [29,46,66]. As expected, the percentage of nitrogen of the triazine framework **CTF-hex6** synthesized under ionothermal reaction conditions is the lowest compared to the synthesized frameworks **CTF-hex1**–**5** due to more defects and more significant decomposition at higher temperatures [25,29,67]. The structure of CTFs from ionothermal reactions with ZnCl**_2_** approaches those of porous carbon materials, especially at temperatures above 400 °C, where a significant amount of nitrogen is lost, such that these CTFs may be better described as nitrogen-doped porous carbon [14,29,34,68]. On the other hand, TFMS-catalysed CTFs usually approach the idealized structure [29]. A clear indication is given by elemental analysis with the significantly higher nitrogen content, i.e., lower nitrogen loss through C–H bond cleavage and carbonization under Brønsted-acid synthesis conditions (Table S2) [25].

**Figure 1:**
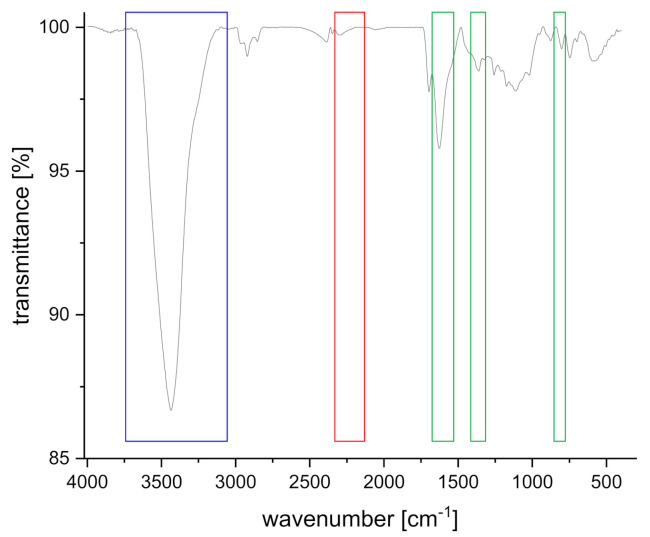
IR spectrum of the synthesized triazine-based framework **CTF-hex6**. In green, at around 1500, 1360, and 810 cm^−1^, the IR bands for triazine units are shown; in red, at around 2200 cm^−1^, there is a small IR band for the nitrile moiety in all spectra, and between 2900 and 3600 cm^−1^, a significant signal for water (in red) is observed.

IR spectroscopic investigations of all frameworks **CTF-hex1**–**6** show a significant amount of water, as seen at the large IR band for water between 2900 and 3600 cm^−1^ (Figure 1 for **CTF-hex6** and Supplementary Information Figures S1–S3 for **CTF-hex1**–**5**, blue). This supports the assumption that one reason for the deviations of the elemental analyses is adsorbed water molecules in the microporous networks during sample preparation. The differences for **CTF-hex6** are probably due to the additional zinc species from the ZnCl_2_ catalyst and porogen. Additionally, the IR spectra show the characteristic C–N stretching and breathing modes for triazine units at around 1500 and 1360 cm^−1^ as well as the breathing modes for the triazine unit at around 810 cm^−1^ (Figure 1 for **CTF-hex6** and Supplementary Information Figures S1–S3 for **CTF-hex1**–**5**, green). Simultaneously, the intense IR bands for the nitrile group at around 2230 cm^−1^ decreased significantly compared to the starting material (Figure 1 for **CTF-hex6** and Supplementary Information Figures S1–S3 for **CTF-hex1**–**5**, red) [27,43,46,68]. These observations prove a successful polymerization, but the presence of the nitrile signal indicates an incomplete conversion and supports the results of the elemental analysis again.

**Figure 2:**
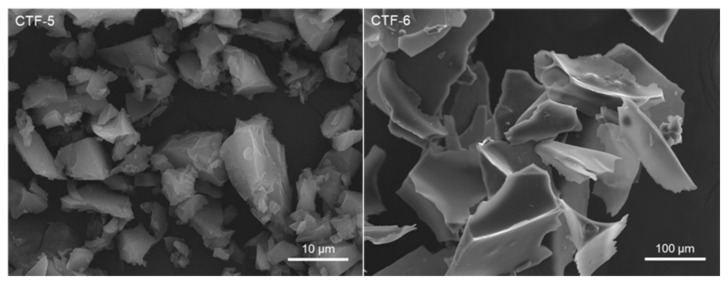
SEM images of the covalent triazine-based frameworks **CTF-hex5** and **-hex6**. The SEM images of the triazine frameworks **CTF-hex1**–**4** are shown in the Supporting Information.

Morphologies of all CTFs were studied by scanning electron microscopy (Figure 2 and Figure S4, Supplementary Information). **CTF-hex1** exhibits a combination of aggregation of spherical particles as well as irregular lumps with different sizes. However, **CTF-hex2**–**5** show the general morphology of aggregates of irregular lumps with different sizes, whereas **CTF-hex6** shows sheet-like morphology.

Powder X-ray diffractograms in Figure S14, Supplementary Information illustrate the expected largely amorphous nature of the CTF-hex materials. The diffractograms for the mixed-linker compounds exhibit three broad bands around 17°, 27°, and 41° 2-theta. For the mono-linker **CTF-hex1** and **CTF-hex6**, the first band occurs already at 2θ ≈ 15° and 11°, respectively, and the band at 17° is not well developed. Noteworthy, the mixed-linker **CTF-hex2**–**5** also features a comparatively sharp peak at 2θ = 17°, which in other mixed-linker work (prepared by the ionothermal route) was assigned to the (111) plane reflection from ZnCl_2_ [46,66]. Obviously, this assignment was not correct, in view of the synthesis of mixed-linker **CTF-hex2**–**5** with the Brønsted acid route by using only trifluoromethanesulfonic acid.

### 3.2. Gas Sorption Studies

**Figure 3:**
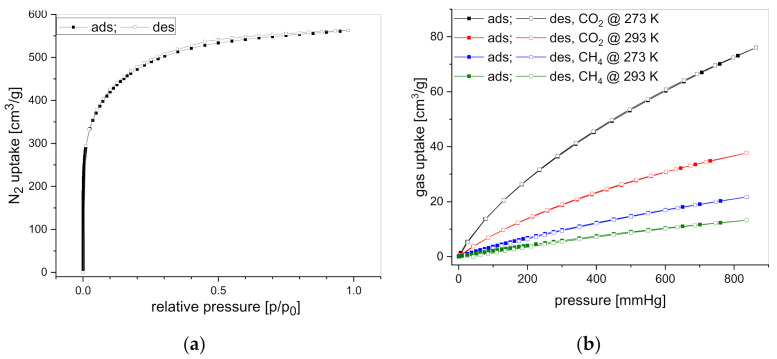
Nitrogen adsorption–desorption isotherms at 77 K (**a**), as well as carbon dioxide and methane uptake at 1 bar (**b**) for triazine framework **CTF-hex6**, are shown exemplarily (closed symbols for adsorption and open symbols for desorption). The other isotherms are shown in Figures S5 and S7 in the Supporting Information.

The porosities of the six synthesized triazine frameworks were characterized by N_2_ sorption measurements at 77 K. Figure 3 shows exemplarily the N_2_ sorption isotherms for **CTF-hex6** (Figure 3a). The N_2_ sorption isotherms of the triazine frameworks **CTF-hex1**–**5** are shown in the Supplementary Information. The Brunauer–Emmett–Teller (BET) surface areas were found to be in the range of 493 m^2^/g to 639 m^2^/g for the **CTFs-hex1**–**5** and 1728 m^2^/g (*p*/*p*_0_ = 0.01–0.05) for the framework **CTF-hex6** via ionothermal reaction conditions (Scheme 1, Table 2). As described before, triazine-based frameworks via ionothermal reaction conditions achieve much higher BET surface areas. One possible explanation for the larger surface area is the decomposition of the triazine moieties because of the high temperature leading to larger pores due to the loss of triazine knots or expanding the structure through the gas formation [14].

The surface area of 1728 m^2^/g for **CTF-hex6** is still at the high end for surface areas for CTFs, which were synthesized at 400 °C under ionothermal conditions. The surface area of CTFs increases with temperature, so CTFs synthesized at, e.g., 600 °C will have higher surface areas [66]. Examples of CTFs with higher surface areas (Table S3, Supplementary Information) are fl-CF-400 to -600 (2862–2113 m^2^/g from 9*H*-fluorene-2,7-dicarbonitrile) [27], PCTF-1 (2235 m^2^/g, from tetrakis(4-cyanophenyl)ethylene) [61,63] or mixed-linker MM1 and MM3 (1800 and 1884 m^2^/g, from the tetranitrile tetrakis(4-cyanophenyl)ethylene (M) with terephthalonitrile (M1), and 4,4′-biphenyldicarbonitrile (M3), respectively) [66].

The isotherm of **CTF-hex6** can be classified as a type Ib isotherm that indicates the framework’s microporous nature (Figure 3a) [69]. The isotherms of **CTF-hex1**–**5** synthesized with TFMS show different behavior than the isotherm of **CTF-hex6** but are similar among each other and can be classified as a combination of isotherm type IV in the lower pressure region and type II at higher relative pressure (Figure S5, Supplementary Information). Mesoporous adsorbents give type IV isotherms due to adsorbent-adsorptive interactions and the interactions between the molecules in the condensed state. Type II isotherms result from unrestricted monolayer multilayer adsorption observed for nonporous or macroporous materials [69]. The adsorption isotherms of **CTF-hex2**–**5** also exhibit hysteresis loops (Figure S5, Supplementary Information). The hysteresis of **CTF-hex2** can be classified as an H3 type of hysteresis, which is generally observed for non-rigid aggregates of plate-like particles and macropores not wholly filled with pore condensate. The triazine frameworks **CTF-hex3**–**5** exhibit H4 type of hysteresis associated with narrow slit-like pores as shown in the Supplementary Information (Figure S5) [69]. The isotherm of **CTF-hex1** is only of type II with H4 type hysteresis. 

Pore size distributions were derived by the density functional theory (DFT) with the ‘carbon slit pore’ model (Section 2.2, Figure S6, Supplementary Information). The micropore (V_0.1_) and total pore volume (V_tot_) was calculated from the N_2_ adsorption isotherms at 77 K. The ratio of V_0.1_/V_tot_ represents the degree of microporosity (Table 2). All CTFs show V_0.1_/V_tot_ values in the range of 0.7–0.8; **CTF-hex2** possesses the highest microporosity with 82%, followed by **CTF-hex5** with 81% (Table 2). 

Typically, the ultramicropores (pores of width < 7 Å) are favorable for CO_2_ capture because small pore size could contribute to a deep overlap of potential and thus strong interaction. Therefore, we calculated ultramicropores from CO_2_ uptake at 273 K (Table 2) as the diffusion of N_2_ molecules at 77 K into ultramicropores is relatively slow (Figure S4, Supplementary Information) [70]. Using CO_2_ to determine ultramicropores ensures a faster equilibration and a slight extension towards the analysis of smaller pores [46,61].

Nitrogen-containing framework structures are known for their excellent CO_2_ uptake capacity because of the quadrupole moment of CO_2_ and the Lewis-basic properties of nitrogen atoms [13,26,46,71,72]. Therefore, we determined the gas uptakes of triazine frameworks **CTF-hex1**–**6** obtained from the respective adsorption isotherms (Figure S4, Supplementary Information) for CO_2_ at 1 bar, as summarized in Table 2. The CO_2_ adsorption of the CTF networks **CTF-hex1**–**6** at 273 K, and 1 bar is in the range of 62–76 cm^3^/g and shows complete reversibility, i.e., a coincidence of the adsorption and desorption branches as shown exemplarily for framework **CTF-hex6** in Figure 3 right (for **CTF-hex1**–**5** see Figure S4, Supplementary Information). This complete reversibility indicates that CO_2_ sorption occurs through unhindered physisorption in predominantly microporous materials.

Among all six CTFs, the mixed-linker triazine framework **CTF-hex4** exhibits the highest CO_2_ adsorption of 76 cm^3^/g (at 273 K) and 48 cm^3^/g (at 293 K) at 1 bar (Table 2, entry 4). This value is higher than that for the pure hexanitrile **CTF-hex1** with 76 cm^3^/g (at 273 K and 1 bar, Table 2, entry 1). The CO_2_ sorption values of **CTF-hex4** are highly comparable to previously reported uptake capacities of CTFs (Table S3, Supplementary Information) [3,25,27,46,64].

In contrast to the nitrogen sorption isotherms, the CO_2_ uptake of framework **CTF-hex6** synthesized via ionothermal reaction is not exceptionally higher than the other frameworks **CTF-hex1**–**5**. It is relatively comparable to the other CO_2_ uptakes. Compared to the pure hexanitrile framework **CTF-hex1** synthesized with TFMS, the framework **CTF-hex6** has a slightly higher CO_2_ uptake of 70 cm^3^/g at 273 K and a slightly lower uptake of 35 cm^3^/g at 293 K.

**Figure 4:**
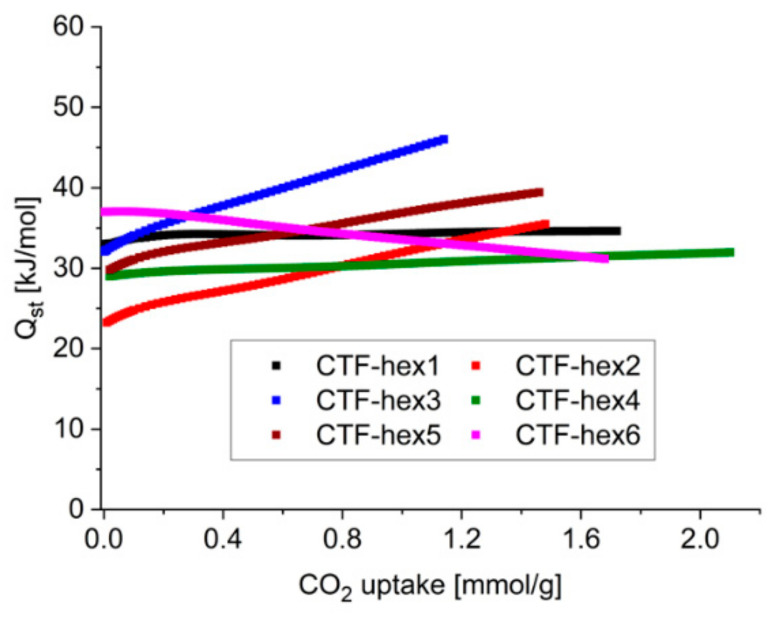
The variation of isosteric heat of adsorption (*Q_st_*) with the amount of CO_2_ adsorbed for **CTF-hex1**–**6**, calculated from a pair of adsorption isotherms measured at 273 K and 293 K using the Virial method [74].

The isosteric heat of CO_2_ adsorption, *Q*_st_, was calculated over the whole adsorption range from the 273 K and 293 K isotherms for CO_2_ in **CTF-hex1**–**6** using the Virial method (Figure 4, see Supplementary Information for details) [73,74]. The different behaviors and physical properties of **CTF-hex6** versus **CTF-hex1** can be explained by a different activation of the nitrile and hence a different number of side-products, unreacted end groups, and remaining reagent traces. 

## 4. Discussion

The heat of adsorption at zero loadings, *Q^0^_st_* is expectedly very similar and between 23 kJ/mol for **CTF-hex5** and 37 kJ/mol for **CTF-hex3**. These values are within the observed range for many CTF materials (Table S2, Supplementary Information). Still, the heats of CO_2_ adsorption remain mostly larger than 25 kJ/mol and, thereby, stay well above the heat of liquefaction of CO_2_ with 17 kJ/mol [36]. A meaningful characterization of porous materials should consider the heat of adsorption over the entire adsorption range (not just at zero coverage). Adsorption usually starts at the sites of the highest binding energy, that is, the heat of adsorption. With the saturation of these sites, then the heat of adsorption decreases. At low coverage, the value of *Q_st_* is determined mainly by the interaction with the strongest binding sites. Hence, CO_2_-interacting functionalities or highly polarising adsorption sites will give the highest *Q_st_* values. 

Minor deviations in *Q_st_* are within the error margin of at least ±3 kJ/mol, which can be assumed on average for *Q_st_* data points [75,76]. Consequently, *Q^0^_st_* values should not be reported or discussed with decimal digits. The calculated increase in *Q_st_* with CO_2_ uptake can be a simultaneous, exothermic process such as the rearrangement of already adsorbed CO_2_ molecules towards a closer, energetically more favorable configuration phase transition material. Binding sites in small channels can cooperatively bind CO_2_ molecules and lead to a slipped-parallel arrangement of CO_2_ molecules by CTF:CO_2_:CO_2_:CTF binding, which gives an extra gain of attraction of about 3 kJ mol^−1^ [71,77]. The CTF:CO_2_:CO_2_:CTF binding interaction correlates with the significant increase in CO_2_ adsorption enthalpy with increasing CO_2_ uptake for **CTF-hex2**, **-hex3**, and **-hex5**. The more typical decrease in *Q_st_* with increased loading of CO_2_ is only seen for **CTF-hex6**. Here, the occupation of binding sites in the order of decreasing binding energies takes place and, at the same time, also indicates an adsorbent with different sites. The different, albeit more typical, *Q_st_* behavior can be explained by the significantly lower ultamicropore volume (V_micro_(CO_2_) in Table 2) together with also a much higher fraction of pores above 20 Å than the **CTF-hex1-5** materials (Figure S6a Supplementary Information). In larger pores, the above-noted CTF:CO_2_:CO_2_:CTF binding interactions cannot take place.

In the case of the adsorption and desorption of CH_4_ of the networks **CTF-hex1** (Figure S4, Supplementary Information) and **CTF-hex6** (Figure 3, right), the pure hexanitrile **CTF-hex6** synthesized with ZnCl_2_ has higher uptake capacities of 20 cm^3^/g at 273 K as well as 12.3 cm^3^/g at 293 K and 1 bar (Table 2, entry 6) than framework **CTF-hex1** (18.1 cm^3^/g at 273 K and 10.2 cm^3^/g at 293 K, Table 2, entry 1). All in all, the CH_4_ uptake capacities were found to be in the range of 17.6–27 cm^3^/g at 273 K and 1 bar. Again, the mixed-linker framework **CTF-hex4** has the highest CH_4_ uptake capacity. Within CTF materials, the reported CO_2_ and CH_4_ uptake capacities correspond to frequently reported values (Table S3, Supplementary Information).

## 5. Conclusions

In summary, we presented the extended, *pseudo*-octahedral 1,4-bis(tris(4′-cyanophenyl)methyl)benzene (BTB-nitrile, **1**) as a new tectone for the synthesis of covalent triazine-based frameworks CTFs. Trimerization reactions among the BTB-nitrile **1** were performed under ionothermal reaction conditions with ZnCl_2_ at 400 °C and under strong Brønsted acid conditions with trifluoromethanesulfonic acid (TFMS) at room temperature. As expected, the framework **CTF-hex6** synthesized via ionothermal reaction conditions exhibited a high BET surface area of 1728 m^2^/g compared to the triazine framework **CTF-hex1** with 557 m^2^/g using milder Brønsted acid conditions. In contrast, the uptake of CO_2_ at 293 K was higher for the structure **CTF-hex1** than for **CTF-hex6**.

Depending on previous work in our group, we performed a mixed-linker approach combining BTB-nitrile **1** with various linkers using TFMS as Brønsted acid. This approach resulted in higher BET surface areas of around 620 m^2^/g for nitrogen adsorption than the pure BTB-based framework **CTF-hex1**. Only the surface area of the triazine framework **CTF-hex3** is in the same range. A possible explanation could be the interpenetration of the framework structure. However, the combinations between BTB-nitrile **1** and 1,3,5-tricyanobenzene (**4**) and tetrakis(4-cyanophenyl)methane (**5**), respectively, yielded framework structures with good CO_2_ and CH_4_ uptake capacities at 273 K. Because of their high stability, the six triazine framework structures **CTF-hex1**–**6** synthesized by exploring the extended, *pseudo*-octahedral nitrile **1** are promising materials mainly for the storage of CO_2_ and CH_4_.

## Figures and Tables

**Table 1:** Monomers and ratio, synthesis method and yields for triazine-based frameworks **CTF-hex1**–**6**.

Entry	Monomer (Molar Ratio)	Framework	Method ^a^	Yield ^b^
1	**1**	**CTF-hex1**	a	84%
2	**1** with **2** (1:3)	**CTF-hex2**	a	46%
3	**1** with **3** (1:3)	**CTF-hex3**	a	50%
4	**1** with **4** (1:2)	**CTF-hex4**	a	65%
5	**1** with **5** (1:0.6)	**CTF-hex5**	a	52%
6	**1**	**CTF-hex6**	b	68%

^a^ **CTF-hex1**–**hex5** was synthesized using TFMS, whereas **CTF-hex6** was synthesized by using ZnCl_2_. ^b^ The calculation of the yield is based on hypothetical 100% polymerization. A hybrid linker approach using TFMS as Brønsted acid was carried out for the first time. The yields were not optimized.

**Table 2:** Porosity data for the covalent triazine-based frameworks **CTF-hex1**–**5** synthesized with TFMS and **CTF-hex6** synthesized via ionothermal reaction conditions and carbon dioxide and methane uptake capacities; the corresponding adsorption–desorption isotherms are shown in the Supplementary Information ^a^.

Entry	CTF-hex	S_BET_^b^ [m^2^/g]	S_Lang_^c^ [m^2^/g]	V_0.1_^d^ [cm^3^/g]	V_tot_^e^ [cm^3^/g]	V_0.1_/V_tot_	V_micro_(CO_2_) ^f^ [cm^3^/g]	CO_2_ (273 K)		CO_2_ (293 K)		*Q*^0^_st_ (CO_2_)	CH_4_ [cm^3^/g]	
								[cm^3^/g]	[mmol/g] ^g^	[cm^3^/g]	[mmol/g] ^h^	[kJ/mol] ^i^	273 K	293 K
1	**1**	557 ^j^	669 ^j^	– ^k^	0.246 ^j^	– ^k^	0.091	64	2.8	40	1.65	33	18.1	10.2
2	**2**	620	680	0.23	0.28	0.82	0.111	58	2.5	33	1.34	23	20	– ^l^
3	**3**	493	626	0.19	0.24	0.79	0.091	56	2.5	26	1.05	32	17.6	– ^l^
4	**4**	609	759	0.23	0.31	0.74	0.118	76	3.4	48	1.96	29	27	– ^l^
5	**5**	638	790	0.25	0.31	0.81	0.101	62	2.7	33	1.35	29	24	– ^l^
6	**6**	1728	2123	0.66	0.87	0.76	0.068	70	3.1	35	1.46	37	20	12.3

^a^ Values were rounded according to the estimated measurement uncertainties. For gas uptakes, this uncertainty is ±5%. The N_2_ gas uptakes are the basis for BET and Langmuir surface areas as well as pore volumes for this then also uncertainties of ±5% can be assumed. This gives, for example, an uncertainty of ±25 m^2^ g^−1^ for surface areas of 500 m^2^ g^−1^ and ±50 m^2^ g^−1^ for surface areas of 1000 m^2^ g^−1^. We note, however, that in the literature, CO_2_ and other gas uptakes are typically given with one decimal digit in the unit cm^3^/g and with two decimal digits in the unit mmol/g, which is more than the underlying measurement accuracy would allow. ^b^ BET surface area derived from the N_2_ adsorption isotherm at 77 K over the relative pressure range *p*/*p*_0_ = 0.01–0.05. ^c^ Langmuir surface area calculated over the ‘extended’ *p*/*p*_0_ range of 0–0.15 for improved averaging and agreement between data. ^d^ Pore volume from N_2_ adsorption isotherm at *p*/*p*_0_ = 0.1 for pores ≤2 nm (20 Å) (micropore volume). ^e^ Total pore volume at *p*/*p*_0_ = 0.95 for pores ≤20 nm. ^f^ Pore volume from the CO_2_ NLDFT model at 273 K for pores with diameters smaller than 1 nm (10 Å) (ultramicropore volume) (cf. Figure S6, Supplementary Information). ^g^ Transformation from cm^3^/g into mmol/g at 273 K: value in [cm^3^/g]: (22.711 cm^3^/mmol) = value in [mmol/g] (22.711 L is the molar volume at 1 bar and 273 K for an ideal gas). ^h^ Transformation from cm^3^/g into mmol/g at 293 K: value in [cm^3^/g]: (24.375 cm^3^/mmol) = value in [mmol/g] (24.375 L is the molar volume at 1 bar and 293 K for an ideal gas). ^i^ The heat of adsorption for CO_2_ at zero loadings (*p*/*p*_0_ = 0.0078) from CO_2_ adsorption isotherms acquired at 273 and 293 K and calculated via the Virial method (see Supplementary Information for details). ^j^ Surface areas were determined several times and obtained BET surface areas depended strongly on preparation and were found to be in the range of 0–557 m^2^/g. ^k^ Because of the results from BET surface determination, no micropore volume was calculated. ^l^ not measured.

## Data Availability

The molecular data are deposited on the chemotion depository.

## Supplementary Materials

The following are available online at https://www.mdpi.com/article/10.3390/ma14123214/s1.
