# Supplementary material for: Covalent Triazine Frameworks Based on the First Pseudo-Octahedral Hexanitrile Monomer via Nitrile Trimerization: Synthesis, Porosity, and CO2 Gas Sorption Properties
Source: Materials (Basel). 2021 Jun 10;14(12):3214. doi: 10.3390/ma14123214 (PMC8230500; doi:10.3390/ma14123214)
Supplement: Supplementary file 1 [file materials-14-03214-s001.zip › materials-1243594-supplementary-for publish.pdf]

materials

MDPI

Supplementary materials

Covalent Triazine Frameworks Based on the First *Pseudo*-Octahedral Hexanitrile Monomer via Nitrile Trimerization: Synthesis, Porosity, and CO_2 Gas Sorption Properties

Isabelle D. Wessely [1], Alexandra M. Schade [1,2], Subarna Dey [3], Asamanjoy Bhunia [4], Alexander Nuhnen [3], Christoph Janiak [3] and Stefan Bräse [1,5,*]

1 Institute of Organic Chemistry (IOC), Karlsruhe Institute of Technology (KIT), Fritz-Haber-Weg 6, D-76131 Karlsruhe, Germany; isabelle.wessely@t-online.de (I.D.W.); alexandra-schade@gmx.de (A.M.S.)
2 Herbstreith & Fox GmbH & Co. KG Pektin-Fabriken, D-75305 Neuenbürg, Germany
3 Institute of Inorganic and Structural Chemistry, Heinrich-Heine-University Düsseldorf, D-40204 Düsseldorf, Germany; subarna.chemie@gmail.com (S.D.); Alexander.Nuhnen@uni-duesseldorf.de (A.N.); janiak@uni-duesseldorf.de (C.J.)
4 Department of Chemistry, Inorganic Chemistry Section, Jadavpur University, Jadavpur, Kolkata 700032, India;
asamanjoy.bhunia@gmail.com
5 Institute of Biological and Chemical Systems (IBCS-FMS), Karlsruhe Institute of Technology (KIT), Hermann-von-Helmholtz-Platz 1, D-76344 Eggenstein-Leopoldshafen, Germany
* Correspondence: stefan.braese@kit.edu or braese@kit.edu; Tel.: +49 721 4 2902

Citation: Wessely, I.D.; Schade, A.M.; Dey, S.; Bhunia, A.; Nuhnen, A.; Janiak, C.; Bräse, S. Covalent Triazine Frameworks Based on the First *Pseudo*-Octahedral Hexanitrile Monomer via Nitrile Trimerization: Synthesis, Porosity, and CO_2 Gas Sorption Properties. *Materials* **2021**, *14*, 3214. https://doi.org/10.3390/ma14123214

Academic Editor: Stefano Agnoli

Received: 17 May 2021
Accepted: 7 June 2021
Published: 10 June 2021

Publisher's Note: MDPI stays neutral with regard to jurisdictional claims in published maps and institutional affiliations.

Copyright: © 2021 by the authors. Submitted for possible open access publication under the terms and conditions of the Creative Commons Attribution (CC BY) license (http://creativecommons.org/licenses/by/4.0/).

1. General remarks

1.1 Analytics and Equipment

Nuclear magnetic resonance spectroscopy (NMR): NMR spectra were recorded on the following spectrometer: **^{1}H NMR**: Bruker AC 250 (250 MHz), Bruker Avance 300 (300 MHz), Bruker Avance 400 (400 MHz); **^{13}C NMR**: Bruker AC 250 (62.5 MHz), Bruker AM 400 (100 MHz); **^{19}F NMR**: Bruker Avance 400 (376 MHz). All spectra were recorded at room temperature. The solvents used were Chloroform-d_1 from Eurisotop. Chemical shifts are expressed in parts per million (ppm, δ) and are referenced to $CHCl_3$ (δ = 7.26 ppm, s, for ^{1}H NMR and δ = 77.0 ppm, t, for ^{13}C NMR). All coupling constants (J) are absolute values, and J values are expressed in Hertz (Hz). The description of signals includes: s = singlet, AA'BB' = hydrogens on a para-substituted phenyl ring. The spectra were analyzed according to the first order. The signal structure in ^{13}C NMR was analyzed by DEPT and is described as follows: + = primary or tertiary C-atom (positive signal), – = secondary C-atom (negative signal), and C_q = quaternary C-atom (no signal).

N_2-Adsorption for CTF-hex1: The specific surface area (BET and Langmuir model), as well as the pore size volumes for CTF-hex1, were determined using a Thermo Scientific™ surfer-gas-adsorption-porosimeter from Thermo Scientific. The sample was degassed under an ultra-high vacuum at elevated temperatures. After weighing, the sample tube was then transferred to the analysis port of the sorption analyzer. N_2 sorption isotherms were measured at 77 K (liquid nitrogen bath). Used N_2 gas was of ultrahigh purity (UHP, grade 5.0, 99.999%). A blank measurement with N_2 was done before analysis.

Gas adsorption: Nitrogen sorption isotherms for **CTF-hex2–5** at 77 K were obtained using a NOVA-4000e instrument within a relative pressure range of 10^{-6}–1.0. DFT calculations for the pore size distribution curves were carried out using the native ASWin 2.03 software from Quantachrome Instruments employing the 'N_2 at 77 K on carbon, slit pore, nonlinear density functional theory (NLDFT) equilibrium' model as well as the 'N_2 at 77 K on carbon, slit pore, quenched solid density functional theory (QSDFT) adsorption branch and equilibrium model, which is favorable for disordered micro/mesoporous

carbon materials. CO_2 and CH_4 (and N_2 for **CTF-hex6**) sorption isotherms were measured using a Micromeritics ASAP 2020 automatic gas sorption analyzer equipped with oil-free vacuum pumps (ultimate vacuum $<10^{-8}$ mbar) and valves, which guaranteed contamination-free measurements. The sample was connected to the preparation port of the sorption analyzer and degassed under vacuum until the outgassing rate, i.e., the rate of pressure rise in the temporarily closed manifold with the connected sample tube, was less than 2 μTorr/min at the specified temperature of 200 °C. After weighing, the sample tube was then transferred to the analysis port of the sorption analyzer. All used gases (H_2, He, N_2, CO_2, CH_4) were of ultrahigh purity (UHP, grade 5.0, 99.999%), and the STP volumes are given according to the NIST standards (293.15 K, 101.325 kPa). Helium gas was used for the determination of the cold and warm free space of the sample tubes. N_2 sorption isotherms were measured at 77 K (liquid nitrogen bath), whereas CO_2 and CH_4 sorption isotherms were measured at 293 ± 1 K (passive thermostat) and 273.15 K (ice/deionized water bath). The DFT pore size distributions from CO_2 ('NLDFT slit pore' model) were calculated using the ASAP 2020 v3.05 software.

Infrared spectroscopy: IR spectra were recorded with an FT-IR Bruker IFS 88 spectrometer with OPUS software using the attenuated total reflection technique (ATR) or the DRIFT technique (diffused reflectance infrared Fourier transform spectroscopy) for solids. The absorption band is given in wavenumbers $\tilde{\upsilon}$ in cm^{-1}. The forms and intensities of the bands were characterized as follows: vs = very strong (0–20% T), s = strong (21–40% T), m = medium (41–60% T), w = weak (61–80% T), vw = very weak (81–100% T, transmission).

Mass spectrometry (EI-MS, FAB-MS, HRMS): The samples were measured either by EI-MS (Electron Ionization Mass Spectrometry) or FAB-MS (Fast Atom Bombardment Mass Spectrometry). EI- and FAB were performed by using Finnigan MAT 90 (70 eV). The molecular fragments were given as mass/charge ratio m/z; the intensity of the signals was given as a percentage of the intensity of the base signal (100%) for the EI mass spectra. **EI-MS:** The abbreviation [M^+] was used for the molecular ion. **FAB-MS:** The abbreviation [M^+ + H] was used for the protonated molecular ion. The following abbreviations were used for the **HRMS** (High-Resolution Mass Spectrometry): calcd. = theoretical value (calculated); found = value obtained by measurement.

Elemental analysis (EA): The measurements were performed on an Elementar Vario MICRO device using a Sartorius M2P precision balance. The following abbreviations were used: calcd. = calculated data, found = measured data.

Analytical thin-layer chromatography (TLC): TLC was carried out on Merck silica gel coated aluminum plates (silica gel 60, F_{254}), detected under UV light at 254 nm.

Scanning electron microscopy (SEM): Scanning electron microscopy (SEM) images were obtained using an ESEM Quanta 400 FEG SEM equipped with a secondary electron detector.

Analytical balance: Weighing and yields were determined with Kern ABS.

UV lamp: A UV lamp from Herolab, type UV-6 S/L, was used to visualize spots on thin-layer chromatography plates.

Ultrasonic bath: An ultrasonic bath from Bendelin, type Sonorex Digitec, was used to dissolve solids and agglomerates better.

Oven: The triazine framework synthesis under ionothermal conditions was performed in a tube furnace LOBA-1200-50-400-1-OW from HTM Reetz GmbH.

Powder X-ray diffraction (PXRD): Diffractograms were obtained with a Bruker D2 Phaser diffractometer using a flat sample holder including a flat silicon, low background sample holder and Cu $K\alpha_1/\alpha_2$ radiation with λ = 1.5418 Å at 30 kV. The measurement covered 2theta angles from 5 to 80° over a time of 2 h, that is, with a scan rate of 0.01°/sec. The flat layer sample holders induced a strong broadening of the beam spot at low angle so that only a fraction of the reflected radiation reaches the detector in this angle range. Subsequently, low relative intensities were usually observed at 2θ angles below 7°.

1.2. Solvents and Reagents

Solvent mixtures are understood as volume/volume. Solvents, reagents, and chemicals were purchased from S*igma-Aldrich, ABCR, Acros Organics,* and *Fisher Scientific*. All solvents, reagents, and chemicals were used as purchased unless stated otherwise. Absolute solvents were purchased from a commercial supplier (absolute DMF (*Acros Organics*, < 50 ppm water), absolute chloroform (*Fischer Scientific*, extra dry over molecular sieves), absolute NMP (*N*-methyl-2-pyrrolidinone, *Fischer Scientific*, <50 ppm water)) 4,4'-dibromo-1,1'-biphenyl, 1,3,5-tribromobenzene und 1,4-dicyanobenzene (**2**) are commercially available.

1.3. Preparative Work

Before the reactions with air or moisture-sensitive reagents were carried out, the glass devices have been dried in an oven and under high. After cooling the glass, apparatuses have been sealed with rubber seals under argon counterflow. Reactions have been executed according to Schlenk-techniques using argon as an inert gas. Liquids were added via plastic syringes and V2A-needles.

Solids were added in pulverized form. Reactions at 0 °C were cooled with a mixture of ice/water.

Solvents were removed at 40 °C with a rotary evaporator. Used solvent mixtures were measured volumetrically. An ultraviolet lamp and the phosphomolybdic acid 10 wt% in ethanol were used for detection.

If not stated otherwise, solutions of inorganic salts are saturated aqueous solutions.

If not otherwise specified, the crude products were purified by flash column chromatography following the concepts of Still et al.[1] using silica gel (SIGMA ALDRICH, pore size 60 Å, particle size 40–63 µm) and sand (calcined and purified with hydrochloric acid) as stationary phase. Solvents were distilled before use or p.a. grade solvents used. Solvent mixtures were prepared individually in terms of volume ratios are given as volumetric. The use of a gradient is indicated in the experimental procedures

2. Synthetic Procedures

2.1. Synthesis of Non-commercially Available Nitrile Tectones

Tetraphenylmethane,[2] tetrakis(4-bromophenyl)methane,[2] 1,4-ditritylbenzene[3] and 1,4-bis(tris(4-bromophenyl)methyl)benzene[3] were synthesized according to literature processes.

4,4′-Dicyano-1,1′-biphenyl (**3**) (Scheme S1): Under an argon atmosphere, 3.00 g (9.62 mmol, 1.00 equiv.) 4,4'-dibromo-1,1'-biphenyl and 3.44 g (38.5 mmol, 4.00 equiv.) copper(I)cyanide in 60 mL DMF were refluxed in a 250 mL Schlenk-flask under vigorous stirring for 2 h. After cooling to room temperature, the solvent was removed under reduced pressure, the residue was filtered off and washed with dichloromethane. The crude product was purified via flash column chromatography (silica gel, 4 cm × 20 cm, cyclohexane/ethyl acetate 90:10 → 60:40) to give 1.11 g (5.44 mmol, 57%) of the product **3** as colorless solid. R_f = 0.71 (cyclohexane/ethyl acetate 6:4).– ^{1}H NMR (400 MHz, $CDCl_3$): δ = 7.78 (AA'BB', *J* = 8.3 Hz, 4 H, 3-*H*, 3'-*H*), 7.70 (AA'BB', *J* = 8.3 Hz, 4 H, 2-*H*, 2'-*H*) ppm.–^{13}C NMR (100 MHz, $CDCl_3$): δ = 143.5 (C_q, 2 C, *C*-1, *C*-1'), 132.6 (+, 4 C, *C*-3, *C*-3'), 128.0 (+, 4 C, *C*-2, *C*-2'), 118.5 (C_q, 2 C, *C*N), 112.4 (C_q, 2 C, *C*-4, *C*-4') ppm.– IR (Drift): $\tilde{\nu}$ = 2224 (w), 1602 (w), 1489 (w), 1395 (w), 1310 (vw), 1779 (w), 1224 (vw), 1005 (vw), 858 (w), 815 (s), 712 (vw), 567 (w), 543 (vw), 458 (vw) cm $^{-1}$. – MS (70 eV, EI), *m*/*z* (%): 204 (100) [M^+], 177 (10) [M^+ – HCN], 150 (3) [M^+ – 2 × HCN], 102 (2) [C_7H_4N].

Analytical data are consistent with the literature.[4]

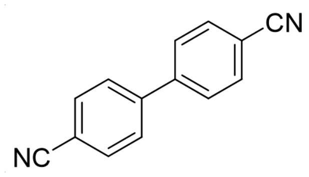

Scheme S1. *4,4′-Dicyano-1,1′-biphenyl (***3***).*

1,3,5-Tricyanobenzene (**4**) (Scheme S2): In a closed 20 mL Vial, 1.00 g (3.18 mmol, 1.00 equiv.) 1,3,5-tribromobenzene, 878 mg (2.39 mmol, 0.750 equiv.) potassium hexacyanoferrate(II), 337 mg (3.18 mmol, 1.00 equiv.) sodium carbonate, 353 mg (0.636 mmol, 0.200 equiv.) 1,1'-bis(diphenylphosphino)ferrocene and 71.4 mg (0.318 mmol, 0.100 equiv.) palladium(II) acetate in 10 mL NMP were refluxed under vigorous stirring for 10 h. After cooling to room temperature, the solvent was removed under reduced pressure, and the residue was purified via flash column chromatography (silica gel, 4 cm × 20 cm, cyclohexane/ethyl acetate 100 → 50:50) to give 72.7 mg (475 µmol, 15%) of the product **4** as colorless solid. R_f = 0.50 (cyclohexane/ethyl acetate 3:1). – ^{1}H NMR (400 MHz, $CDCl_3$): δ = 8.17 (s, 3 H, 2-*H*, 4-*H*, 6-*H*) ppm. – ^{13}C NMR (100 MHz, $CDCl_3$): δ = 138.7 (+, 3 C, *C*H), 116.0 (C_q, 3 C, *C*N), 114.6 (C_q, 3 C, *C*-1, *C*-3, *C*-5) ppm. – IR (Drift): $\tilde{\nu}$ = 3069 (vw), 2924 (vw), 2238 (vw), 1767 (vw), 1694 (w), 1589 (vw), 1426 (w), 1385 (vw), 1301 (vw), 1279 (vw), 1116 (vw), 942 (vw), 893 (w), 815 (vw), 711 (vw), 681 (w), 545 (vw), 503 (vw), 472 (vw), 410 (vw) cm ^{1}. – MS (70 eV, EI), *m*/*z* (%): 153 (100) [M^+], 128 (38) [$C_8H_3N_2$ + H^+].

Analytical data are consistent with the literature.[5]

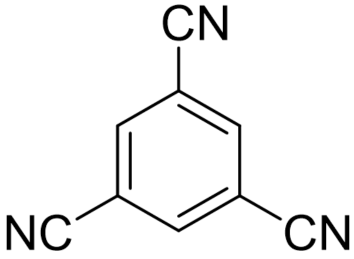

Scheme S2. *1,3,5-Tricyanobenzene (***4***)*

Tetrakis(4-cyanophenyl)methane (**5**) (Scheme S3): Under an argon atmosphere, 750 mg (1.18 mmol, 1.00 equiv.) tetrakis(4-bromophenyl)methane and 1.06 g (11.8 mmol, 10.0 equiv.) copper(I)cyanide in 72 mL DMF were refluxed in a 100 mL Schlenk-flask under vigorous stirring for 3 d. After cooling to room temperature, the solvent was removed under reduced pressure, and the residue was purified via flash column chromatography (silica gel, 4 cm × 20 cm, cyclohexane/ethyl acetate 3:1 → 2:1) to give 135 mg (321 µmol, 27%) of the product **5** as colorless solid. R_f = 0.30 (cyclohexane/ethyl acetate 2:1). – ^{1}H NMR (300 MHz, $CDCl_3$): δ = 7.65 (AA'BB', *J* = 8.7 Hz, 8 H, 3-*H*), 7.29 (AA'BB', *J* = 8.7 Hz, 8 H, 2-*H*) ppm.

The analytical data are consistent with the literature.[6]

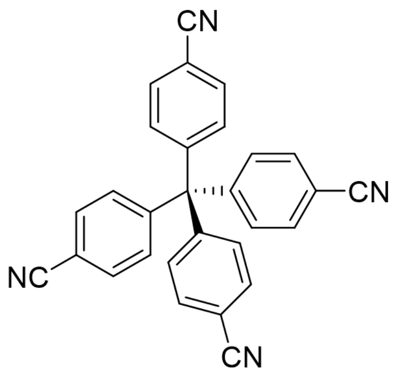

Scheme S3. *Tetrakis(4-cyanophenyl)methane (**5**)*

1,4-Bis(tris(4′-cyanophenyl)methyl)benzene (**1**) (Scheme S4): Unter argon atmosphere 3.00 g (2.90 mmol, 1.00 equiv.) 1,4-bis(tris(4-bromophenyl)methyl)benzene and 6.22 g (69.5 mmol, 24.0 equiv.) copper(I)cyanide in 100 mL DMF were refluxed in a 250 mL Schlenk-flask under vigorous stirring for 5 d. After cooling to room temperature, the solvent was removed under reduced pressure, and 150 mL of a concentrated, aqueous ammonia solution was added. After stirring overnight at room temperature, the aqueous layer was extracted with 3 × 100 mL of dichloromethane, the combined organic layers dried over sodium sulfate, and the solvent was removed under reduced pressure. The residue was purified via flash column chromatography (silica gel, 4 cm × 20 cm, cyclohexane/ ethyl acetate 100 → 60:40) to give 567 mg (795 µmol, 28%) of the product **1** as colorless solid. mp: 383 °C. – R_f = 0.42 (cyclohexane/ethyl acetate 6:4). – ^{1}H NMR (400 MHz, $CDCl_3$): δ = 7.62 (AA'BB', *J* = 8.4 Hz, 12 H, 3'-*H*), 7.25 (AA'BB', *J* = 8.4 Hz, 12 H, 2'-*H*), 7.07 (s, 4 H, 2-*H*, 3-*H*) ppm. – ^{13}C NMR (100 MHz, $CDCl_3$): δ = 149.2 (C_q, 6 C, *C*-1'), 142.8 (C_q, 2 C, *C*-1, *C*-4), 132.2 (+, 12 C, *C*-3'), 131.2 (+, 12 C, *C*-2'), 130.6 (+, 4 C, *C*-2, *C*-3), 118.0 (C_q, 6 C, *C*N), 111.5 (C_q, 6 C, *C*-4'), 65.1 (C_q, 2 C, *C*(Ar)$_4$) ppm. – IR (Drift): $\tilde{\nu}$ = 3036 (vw), 2227 (vw), 1713 (vw), 1602 (vw), 1499 (vw), 1411 (vw), 1218 (vw), 1019 (vw), 910 (vw), 823 (w), 764 (vw), 729 (w), 647 (vw), 594 (vw), 578 (w), 556 (vw) cm ^{1}. – MS (FAB, 3-NBA), *m/z* (%): 713 (100) [M + H^+], 610 (84) [M^+ – C_7H_4N].

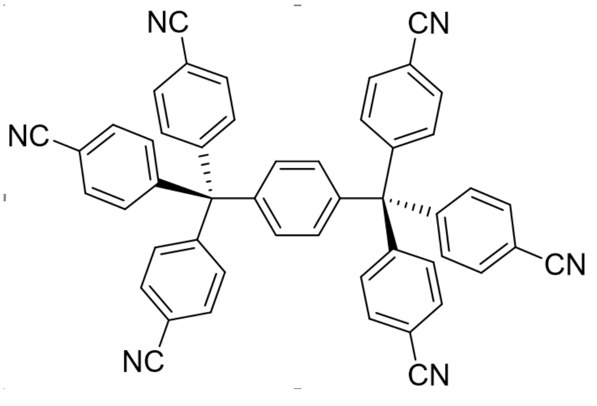

Scheme S4. *1,4-Bis(tris(4′-cyanophenyl)methyl)benzene (**1**)*

2.2. Synthesis of Covalent Triazine-Based Frameworks (CTFs) via Trimerization of Aromatic Nitriles with Trifluoromethanesulfonic acid [7]

General procedure (GP): Under argon atmosphere in a closed 20 mL vial, abs. trifluoromethanesulfonic acid and abs. chloroform (3 mL) was cooled to 0 °C. At this temperature, HPX-nitrile **1** (1.00 eq) and the respective aryl nitrile linker (3.00 eq for di- **2** and **3**, 2.00 eq for tri-**4,** and 0.600 eq for tetratopic linker **5**) dissolved in 10 mL abs. chloroform was added over 30 min. The mixture was stirred for another 2 h at 0 °C and afterward at room temperature overnight. Then, the reaction mixture was poured on a water/$NH_{3(aq)}$-

mixture (100 mL, 20:1) and stirred at room temperature for an additional 2 h. The precipitate was filtered off, washed with distilled water (3 × 10 mL), ethanol (3 × 10 mL), acetone (3 × 10 mL), and chloroform (3 × 10 mL) and dried under high vacuum at 120 °C for 2 d.

'HPX-Nitril' Covalent Triazine-Based Framework (**CTF-hex1**) (Scheme S5): According to GP, 113 µL (194 mg, 1.30 mmol, 8.10 equiv.) trifluoromethane-sulfonic acid and abs. chloroform (3 mL) was cooled to 0 °C. At this temperature, 114 mg (160 µmol, 1.00 equiv.) HPX-nitrile **1** dissolved in abs. chloroform (10 mL) was added over 30 min. The mixture was stirred for another 2 h at 0 °C and afterward at room temperature overnight. After purification and drying under high vacuum (2 d at 120 °C and 10^{-6} mbar) 95.9 mg (134 µmol, 84%) of the product **CTF-hex1** were obtained as slightly yellow solid. IR (ATR): $\tilde{\upsilon}$ = 3370 (vw), 2928 (vw), 2228 (vw), 1602 (vw), 1498 (w), 1406 (vw), 1361 (vw), 1279 (vw), 1191 (vw), 1016 (vw), 810 (vw), 759 (vw), 639 (vw), 559 (vw) cm^{-1}. – EA ($C_{50}H_{28}N_6$): calcd. C 84.25, H 3.96, N 11.79, found C 71.42, H 4.19, N 9.27, S 0.91. –S_{BET} = 557 m^2/g. – $S_{Langmuir}$ = 669 m^2/g. – $V_{Total, Pore}$ = 0.246 cm^3/g. (Determined surface area strongly depends on sample preparation. Measurements were performed on Thermo Scientific™ Surfer.)

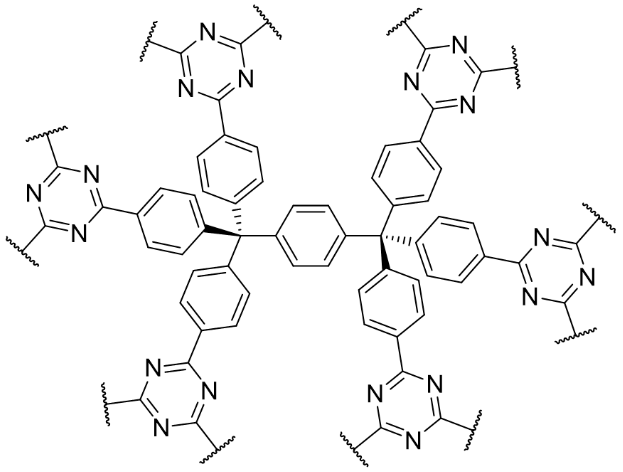

Scheme S5. *'HPX-Nitril' Covalent Triazine-Based Framework* ***CTF-hex1***

'Mono-Aryl-HPX-Nitrile' Covalent Triazine-Based Framework (**CTF-hex2**) (Scheme S6): According to GP, 66.4 µL (114 mg, 757 µmol, 10.8 equiv.) trifluoromethanesulfonic acid and abs. chloroform (3 mL) was cooled to 0 °C. At this temperature, 50.0 mg (70.1 µmol, 1.00 equiv.) HPX-nitrile **1** and 26.9 mg (210 µmol, 3.00 equiv.) 4,4'-dicyanobenzene (**2**) dissolved in abs. chloroform (10 mL) was added over 30 min. The mixture was stirred for another 2 h at 0 °C and afterward at room temperature overnight. After purification and drying under high vacuum (2 d at 120 °C and 10^{-6} mbar) 35.6 mg (32.5 µmol, 46%) of the product **CTF-hex2** were obtained as slightly yellow solid. IR (ATR): $\tilde{\upsilon}$ = 3369 (vw), 2228 (vw), 1604 (vw), 1499 (w), 1407 (vw), 1360 (vw), 1017 (vw), 811 (w), 766 (vw), 549 (vw), 453 (vw) cm^{-1}. – EA ($C_{74}H_{40}N_{12}$): calcd. C 81.01, H 3.67, N 15.32, found C 65.49, H 3.96, N 8.79, S 0.23. – S_{BET} = 620 m^2/g. – $S_{Langmuir}$ = 680 m^2/g. – $V_{Total, Pore}$ = 0.28 cm^3/g.

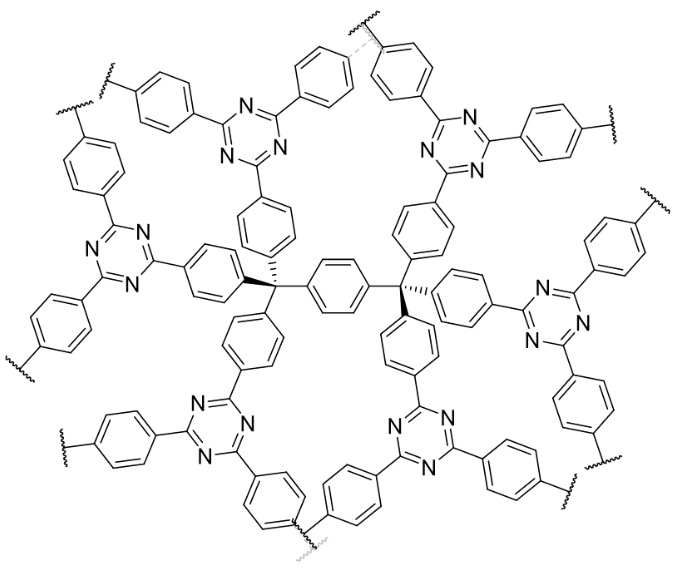

Scheme S6. *′Mono-Aryl-HPX-Nitrile′ Covalent Triazine-Based Framework* ***CTF-hex2***

′Bi-Aryl-HPX-Nitrile′ Covalent Triazine-Based Framework (**CTF-hex3**) (Scheme S7): According to GP, 66.4 µL (114 mg, 757 µmol, 10.8 equiv.) trifluoromethanesulfonic acid and abs. chloroform (3 mL) was cooled to 0 °C. At this temperature, 50.0 mg (70.1 µmol, 1.00 equiv.) HPX-nitrile **1** and 42.9 mg (210 µmol, 3.00 equiv.) 4,4'-dicyanobiphenyl (**3**) dissolved in abs. chloroform (10 mL) was added over 30 min. The mixture was stirred for another 2 h at 0 °C and afterward at room temperature overnight. After purification and drying under high vacuum (2 d at 120 °C and 10^{-6} mbar) 46.2 mg (35.1 µmol, 50%) of the product **CTF-hex3** were obtained as slightly yellow solid.

IR (ATR): $\tilde{\nu}$ = 3308 (vw), 2228 (vw), 1604 (vw), 1500 (w), 1360 (w), 1017 (vw), 809 (w), 766 (vw), 502 (vw) cm^{-1}. – EA ($C_{92}H_{52}N_{12}$): calcd. C 83.37, H 3.95, N 12.68, found C 66.80, H 4.03, N 9.15, S 0.19. – S_{BET} = 493 m^2/g. – $S_{Langmuir}$ = 626 m^2/g. – $V_{Total, Pore}$ = 0.24 cm^3/g.

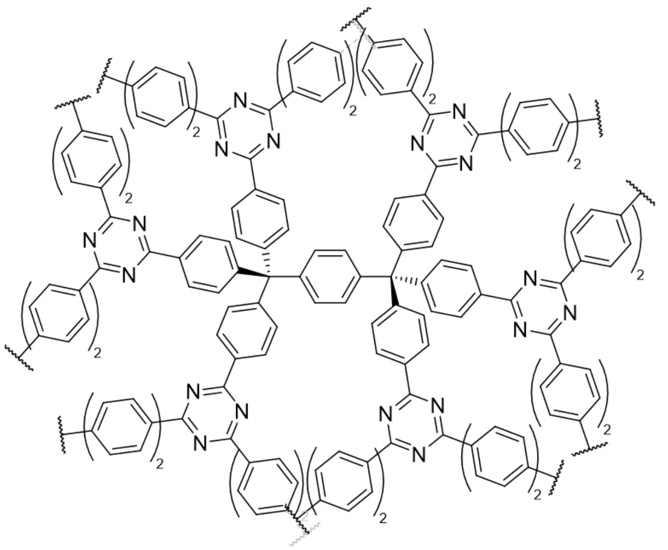

Scheme S7. *′Bi-Aryl-HPX-Nitrile′ Covalent Triazine-Based Framework* ***CTF-hex3***

′Tricyanobenzol-HPX-Nitrile′ Covalent Triazine-Based Framework (**CTF-hex4**) (Scheme S8): According to GP, 75.1 µL (128 mg, 655 µmol, 12.2 equiv.) trifluoromethanesulfonic acid and abs. chloroform (3 mL) was cooled to 0 °C. At this temperature, 50.0 mg (70.1 µmol, 1.00 equiv.) HPX-nitrile **1** and 21.5 mg (140 µmol, 2.00 equiv.) 1,3,5-tricyanobenzene (**4**) dissolved in abs. chloroform (10 mL) was added over 30 min. The mixture was stirred

for another 2 h at 0 °C and afterward at room temperature overnight. After purification and drying under high vacuum (2 d at 120 °C and 10^{-6} mbar) 40.2 mg (39.4 µmol, 65%) of the product **CTF-hex4** were obtained as slightly yellow solid.

IR (ATR): $\tilde{\nu}$ = 3341 (vw), 2229 (vw), 1603 (vw), 1504 (w), 1359 (vw), 1153 (vw), 1016 (vw), 811 (vw), 767 (vw), 501 (vw) cm^{-1}. – EA ($C_{68}H_{34}N_{12}$): calcd. C 80.14, H 3.36, N 16.49, found C 68.26, H 4.11, N 10.60, S 0.48. – S_{BET} = 609 m^2/g. – $S_{Langmuir}$ = 759 m^2/g. – $V_{Total, Pore}$ = 0.31 cm^3/g.

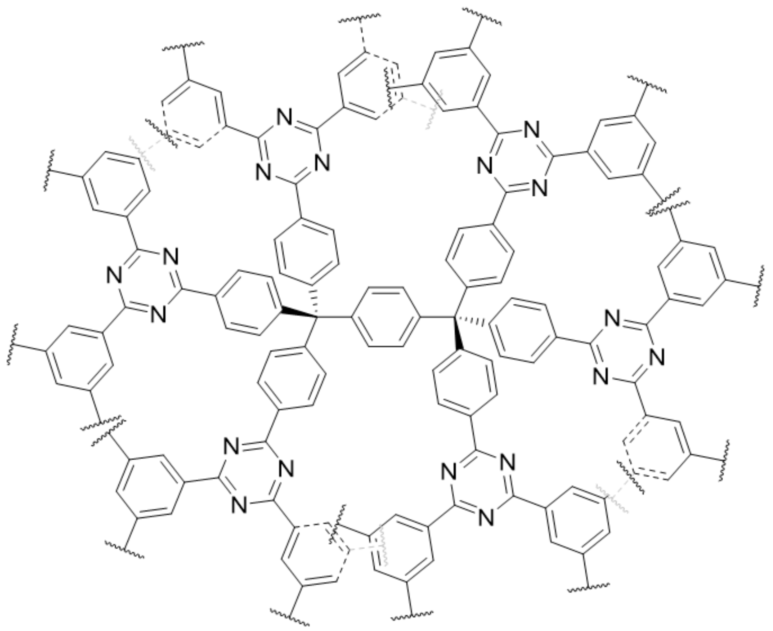

Scheme S8. *'Tricyanobenzol-HPX-Nitrile' Covalent Triazine-Based Framework* ***CTF-hex4***

'TPM-HPX-Nitril' Covalent Triazine-Based Framework (**CTF-hex5**) (Scheme S9): According to GP, 83.1 µL (142 mg, 946 µmol, 13.5 equiv.) trifluoromethanesulfonic acid and abs. chloroform (3 mL) was cooled to 0 °C. At this temperature, 50.0 mg (70.1 µmol, 1.00 equiv.) HPX-nitrile **1** and 17.7 mg (42.1 µmol, 0.600 equiv.) Tetrakis(4-cyanophenyl)methane (**5**) dissolved in abs. chloroform (10 mL) was added over 30 min. The mixture was stirred for another 2 h at 0 °C and afterward at room temperature overnight. After purification and drying under high vacuum (2 d at 120 °C and 10^{-6} mbar), 35.4 mg (36.7 µmol, 52%) of the product **CTF-hex5** were obtained as slightly yellow solid.

IR (ATR): $\tilde{\nu}$ = 3369 (vw), 2228 (vw), 1603 (vw), 1499 (w), 1363 (vw), 1193 (vw), 1016 (vw), 810 (w), 554 (vw) cm^{-1}. – EA ($C_{67.4}H_{37.6}N_{8.4}$): calcd. C 84.35, H 3.91, N 11.74, found C 63.70, H 4.43, N 8.17, S 0.43. – S_{BET} = 638 m^2/g. – $S_{Langmuir}$ = 790 m^2/g. – $V_{Total, Pore}$ = 0.31 cm^3/g.

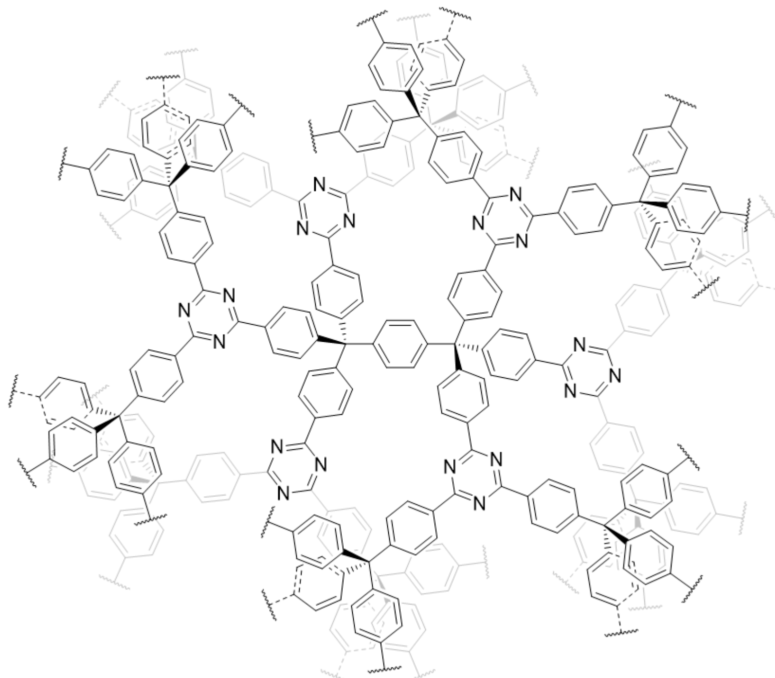

Scheme S9. *'TPM-HPX-Nitril' Covalent Triazine-Based Framework **CTF-hex5***

2.3. Synthesis of Covalent Triazine-Based Frameworks (CTFs) via Trimerization of Aromatic Nitriles using Ionothermal Conditions

'HPX-Nitrile' Covalent Triazine-Based Framework (**CTF-hex6**) (Scheme S10): A mixture of 88.0 mg (123 μmol, 1.00 equiv.) HPX-nitrile **1** and 168 mg (1.23 mmol, 10.0 equiv.) dry zinc chloride were heated in an oven up to 400 °C in a pyrex® ampule (3 × 120 mm) for 42 h. After cooling to room temperature, the ampule was opened carefully. The solid residue was washed with water (200 mL), stirred in diluted HCL (15 mL) overnight, and filtered as well as washed with water (3 × 10 mL) and tetrahydrofuran (3 × 10 mL). After drying under a high vacuum (150 °C and 10^{-6} mbar), 59.4 mg (83.6 μmol, 68%) of the product **CTF-hex6** were obtained as black solid.

IR (KBr): $\tilde{\upsilon}$ = 3435 (vw), 2961 (vw), 2921 (vw), 2853 (vw), 2387 (vw), 2347 (vw), 2297 (vw), 1696 (vw), 1626 (vw), 1361 (vw), 1324 (vw), 1257 (vw), 1211 (vw), 1173 (vw), 1112 (vw), 1021 (vw), 877 (vw), 802 (vw), 747 (vw), 700 (vw), 591 (vw) cm^{-1}. – EA ($C_{50}H_{28}N_6$): calcd. C 84.25, H 3.96, N 11.79, found C 82.19, H 3.08, N 3.58, S 0.46. – S_{BET} = 1728 m^2/g. – $S_{Langmuir}$ = 2123 m^2/g. – $V_{Total, Pore}$ = 0.87 cm^3/g.

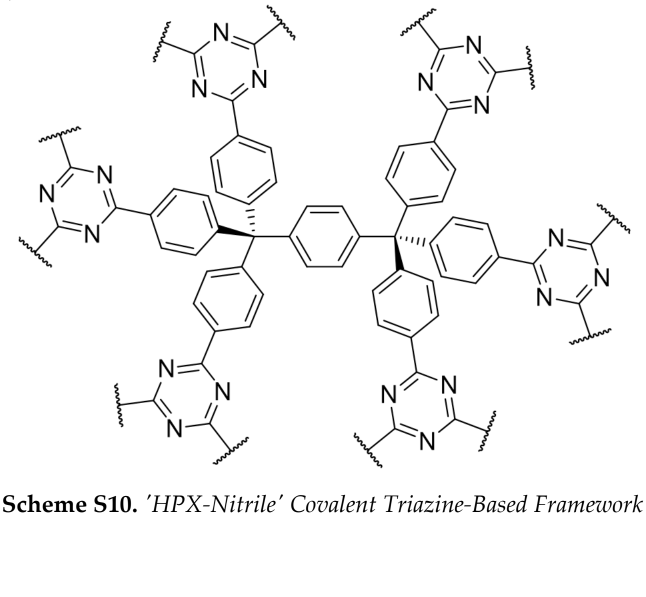

Scheme S10. *'HPX-Nitrile' Covalent Triazine-Based Framework **CTF-hex6***

3. Elemental Analysis

It is only possible to give the molecular weight of the sum formula or repeat unit for the ideal structure of each CTF, assuming 100% polymerization (Table S1).

Table S1. Synthesized triazine-based frameworks **CTF-hex1–6** with monomers **1–5** and their sum formula and molecular weights for the idealized structures.

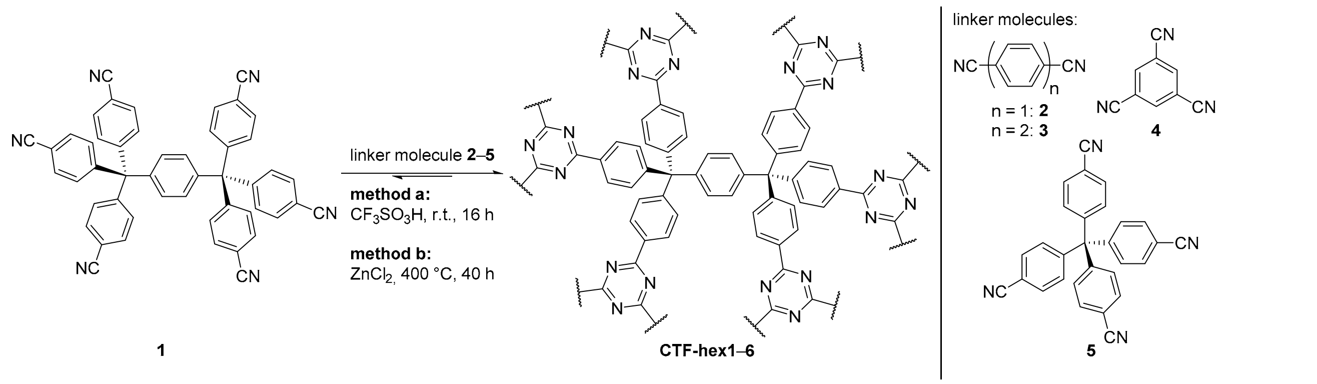

1 CTF-hex1–6 5

Framework [a]	Monomer (Molar Ratio)	Monomer Sum Formula	CTF Sum Formula [b]	Molecular Weight [g/mol] [b]
**CTF-hex1**	**1**	$C_{50}H_{28}N_6$	$C_{50}H_{28}N_6$	712.82
**CTF-hex2**	**1** with **2** (1:3)	$C_{50}H_{28}N_6$, $C_8H_4N_2$ (× 3)	$C_{74}H_{40}N_{12}$	1097.22
**CTF-hex3**	**1** with **3** (1:3)	$C_{50}H_{28}N_6$, $C_{14}H_8N_2$ (× 3)	$C_{92}H_{52}N_{12}$	1325.51
**CTF-hex4**	**1** with **4** (1:2)	$C_{50}H_{28}N_6$, $C_9H_3N_3$ (× 2)	$C_{68}H_{34}N_{12}$	1019.10
**CTF-hex5**	**1** with **5** (1:0.6)	$C_{50}H_{28}N_6$, $C_{29}H_{16}N_4$ (× 0.6)	$C_{67.4}H_{37.6}N_{8.4}$	965.10
**CTF-hex6**	**1**	$C_{50}H_{28}N_6$	$C_{50}H_{28}N_6$	712.82

[a] **CTF-hex1** to **–hex5** was synthesized by using TFMS, whereas **CTF-hex6** was synthesized by using $ZnCl_2$. [b] Calculation of the yield is based on hypothetical 100% polymerization.

The repeat units are as follows:

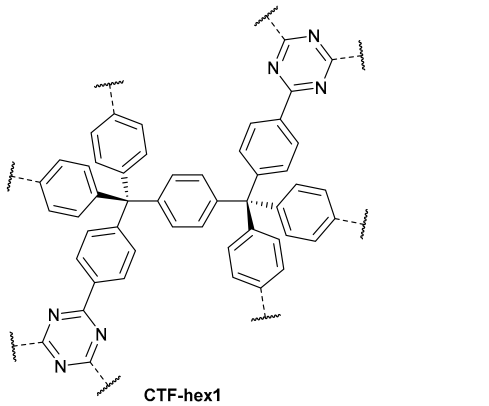

CTF-hex1
Chemical Formula: $C_{50}H_{28}N_6^{8\bullet}$
Molecular Weight: 712.82 g/mol
Elemental Analysis: C, 84.25; H, 3.96; N, 11.79%

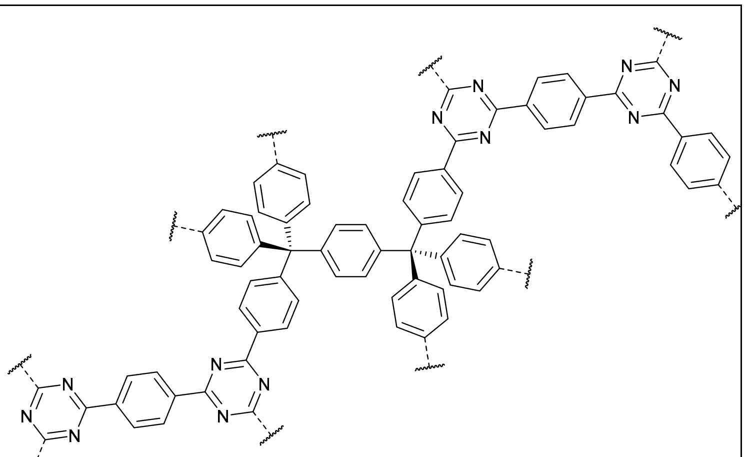

CTF-hex2
Chemical Formula: $C_{74}H_{40}N_{12}^{10\bullet}$
Molecular Weight: 1097.22 g/mol
Elemental Analysis: C, 81.01; H, 3.67; N, 15.32%

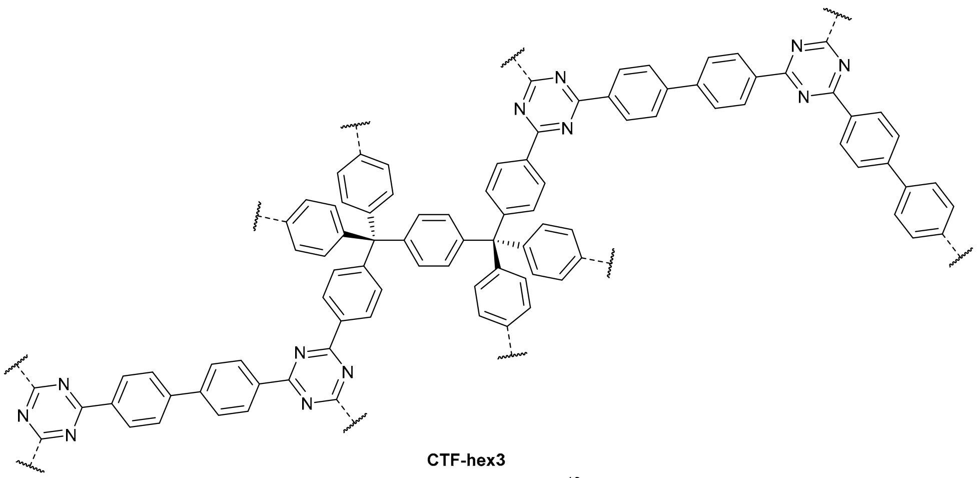

CTF-hex3
Chemical Formula: $C_{92}H_{52}N_{12}{}^{10\bullet}$
Molecular Weight: 1325.51 g/mol
Elemental Analysis: C, 83.36; H, 3.95; N, 12.68%

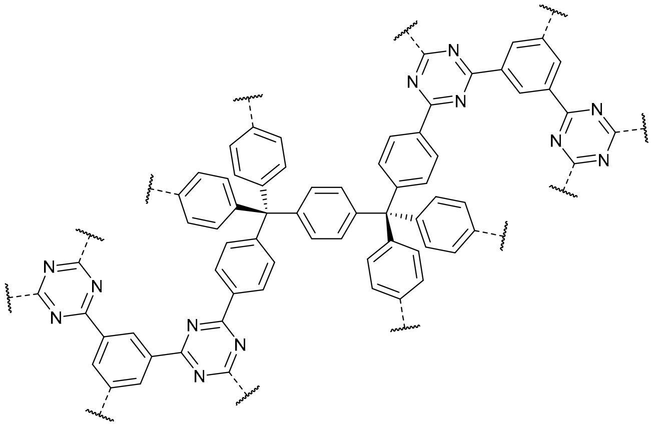

CTF-hex4
Chemical Formula: $C_{68}H_{34}N_{12}{}^{12\bullet}$
Molecular Weight: 1019.10 g/mol
Elemental Analysis: C, 80.14; H, 3.36; N, 16.49%

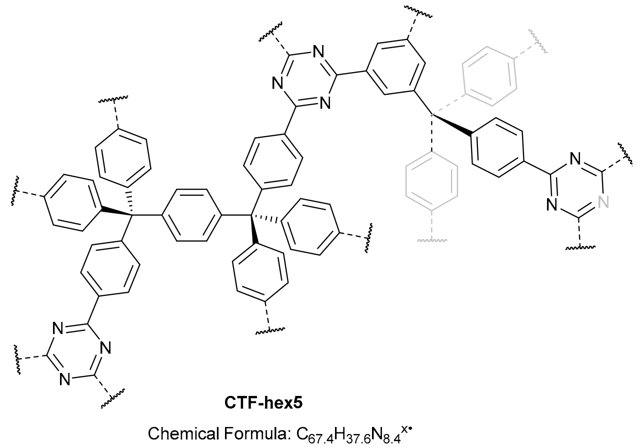

CTF-hex5
Chemical Formula: $C_{67.4}H_{37.6}N_{8.4}{}^{x\bullet}$
Molecular Weight: 965.10 g/mol
Elemental Analysis: C, 84.35; H, 3.91; N, 11.74%

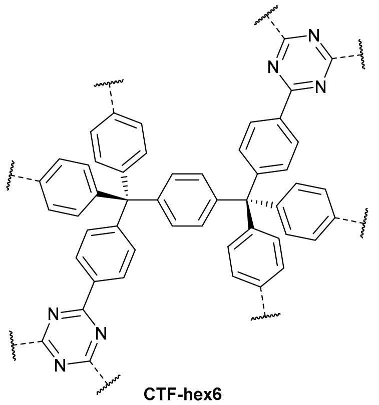

CTF-hex6
Chemical Formula: $C_{50}H_{28}N_{6}{}^{8\bullet}$
Molecular Weight: 712.82 g/mol
Elemental Analysis: C, 84.25; H, 3.96; N, 11.79%

Table S2. Summary of the elemental analyses of the synthesized triazine-based frameworks **CTF-hex1–6** as well as the C/H- and C/N-ratios for a better comparison.

Entry	Framework	Calculated (%) [a]					Found (%)				
		C [b]	H [b]	N [b]	C/H [c]	C/N [c]	C [b]	H [b]	N [b]	C/H [c]	C/N [c]
1	**CTF-hex1**	84.25	3.96	11.79	1.77	8.34	71.42	4.19	9.27	1.42	8.99
2	**CTF-hex2**	81.01	3.67	15.32	1.84	6.17	65.49	3.96	8.79	1.38	8.69
3	**CTF-hex3**	83.37	3.95	12.68	1.76	7.67	66.80	4.03	9.15	1.38	8.52
4	**CTF-hex4**	80.14	3.36	16.49	1.99	5.67	68.26	4.11	10.60	1.38	7.51
5	**CTF-hex5**	84.35	3.91	11.74	1.80	8.38	63.70	4.43	8.17	1.20	9.10
6	**CTF-hex6**	84.25	3.96	11.79	1.77	8.34	82.19	3.08	3.58	2.22	26.78

[a] Calculation based on the used stoichiometric ratios without considering the adsorption of moisture; [b] data are in wt%; [c] ratio of molar percentage.

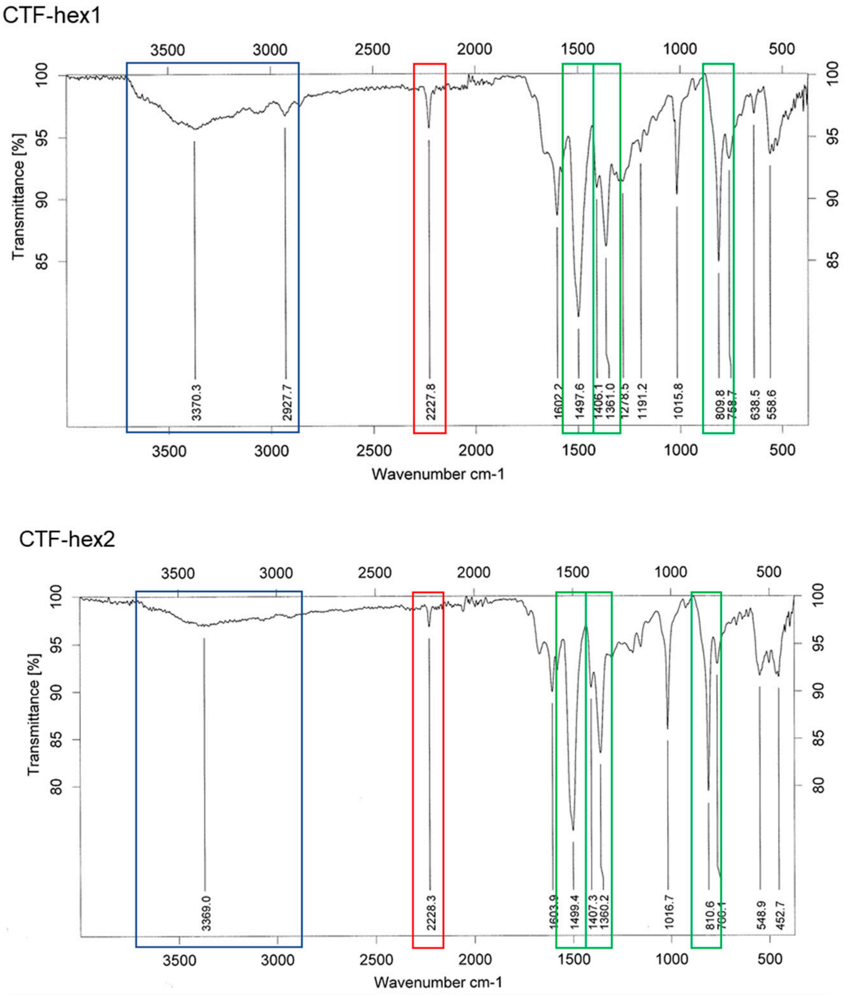

Figure S1. IR spectra of the triazine-based framework structures **CTF-hex1** and **CTF-hex2**. In green at around 1500, 1360, and 810 cm$^{-1,}$ the IR bands for triazine units are shown; in red at around 2230 cm^{-1} is a small IR band for the nitrile moiety in all spectra and between 2900 and 3600 cm^{-1} a significant signal for water is observed (blue).

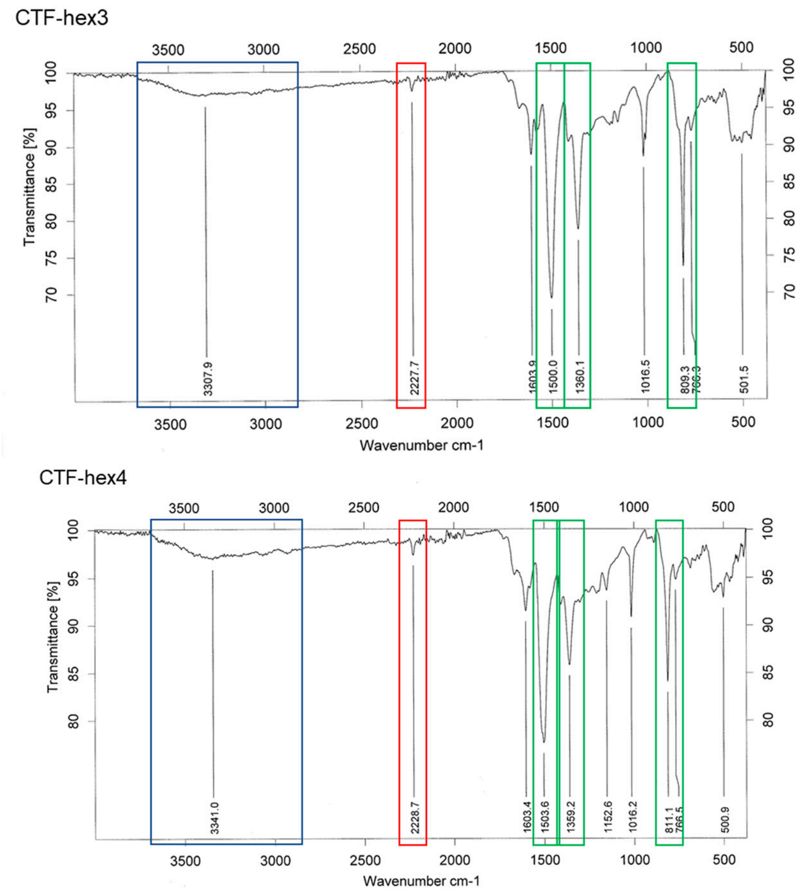

Figure S2. IR spectra of the triazine-based framework structures **CTF-hex3** and **CTF-hex4**. In green at around 1500, 1360, and 810 cm^{-1}, the IR bands for triazine units are shown; in red at around 2230 cm^{-1} is a small IR band for the nitrile moiety in all spectra and between 2900 and 3600 cm^{-1} a significant signal for water is observed (blue).

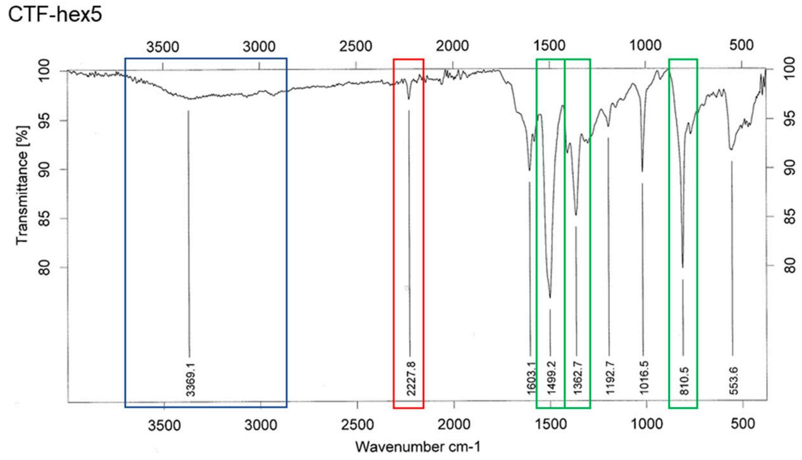

Figure S3. IR spectrum of the triazine-based framework structure **CTF-hex5**. In green at around 1500, 1360, and 810 cm^{-1}, the IR bands for triazine units are shown; in red at around 2230 cm^{-1} is a small IR band for the nitrile moiety in all spectra and between 2900 and 3600 cm^{-1} a significant signal for water is observed (blue).

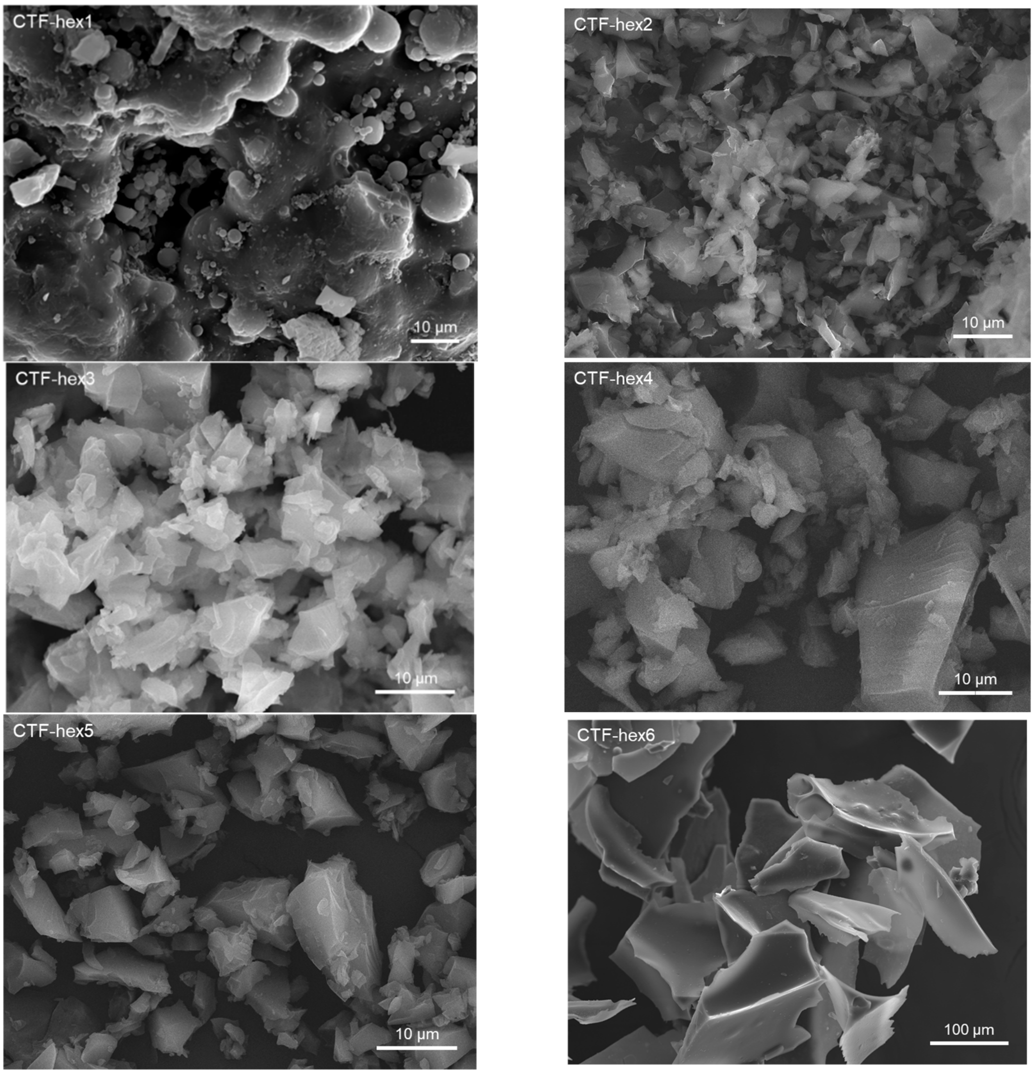

Figure S4. SEM images of the covalent triazine-based frameworks **CTF-hex1–6** (labels are given in the upper left corner of each image).

4. Gas sorption Measurements

4.1. Nitrogen Adsorption-desorption Isotherms

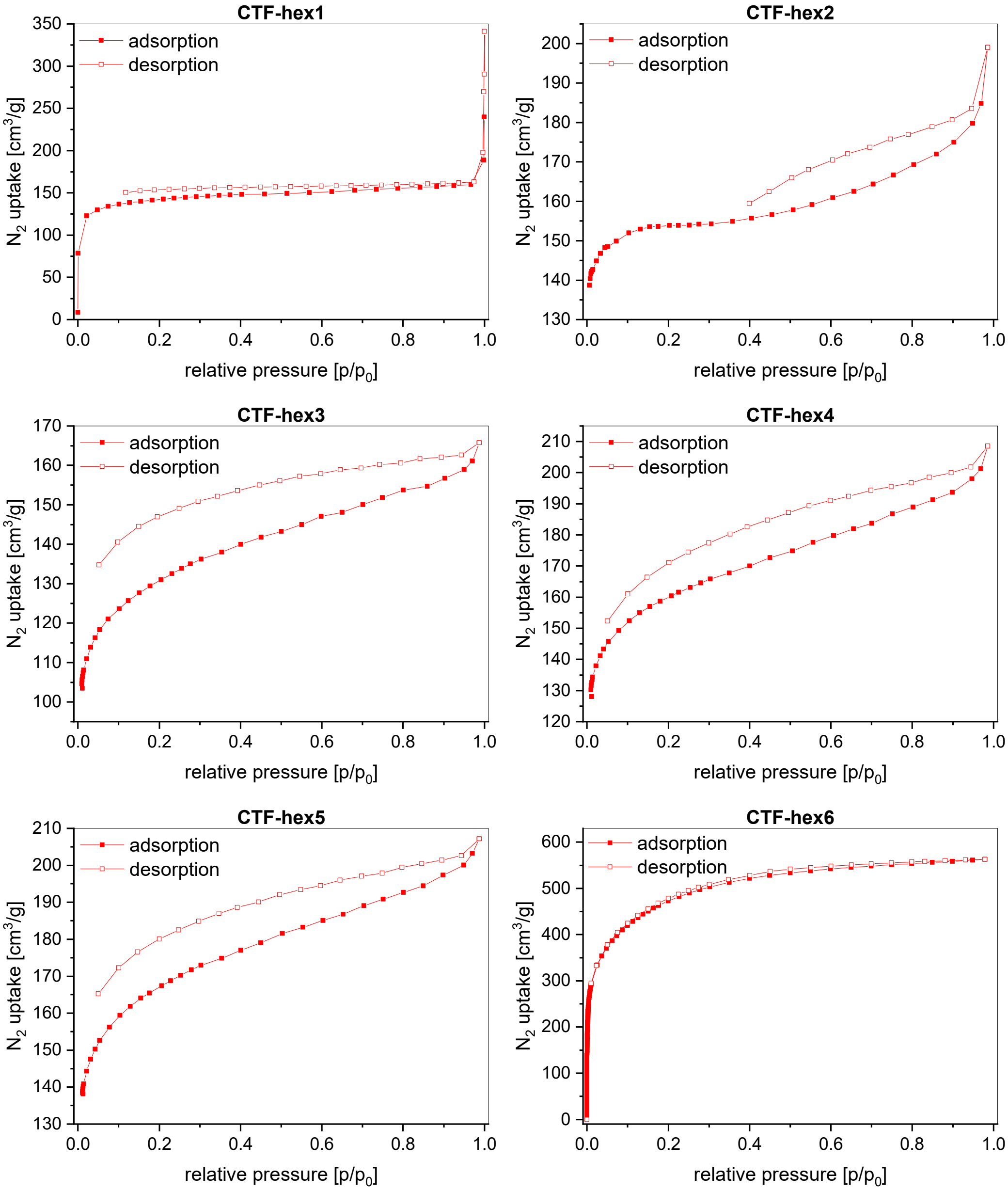

Figure S5. Nitrogen adsorption-desorption isotherms at 77 K (closed symbols for adsorption and open symbols for desorption) for **CTF-hex1–6**. **CTF-hex2–5** were measured on a NOVA-4000e instrument. **CTF-hex1** was measured on a Thermo Scientific™ adsorption porosimeter, and the surface depends strongly on the preparation conditions. **CTF-hex6** was measured using a Micromeritics ASAP 2020.

Note that the desorption branch has not closed for **CTF-hex2–5** due to the set time constraints during the measurement. The used instrument NOVA 4000e was set for short measurements due to high demand.

4.2. Pore size distributions

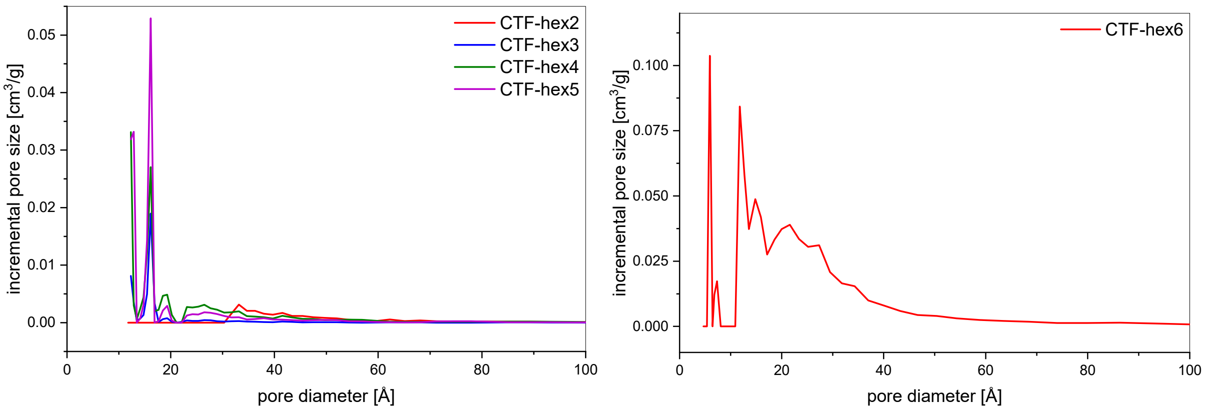

(**a**) from N_2 sorption, NLDFT

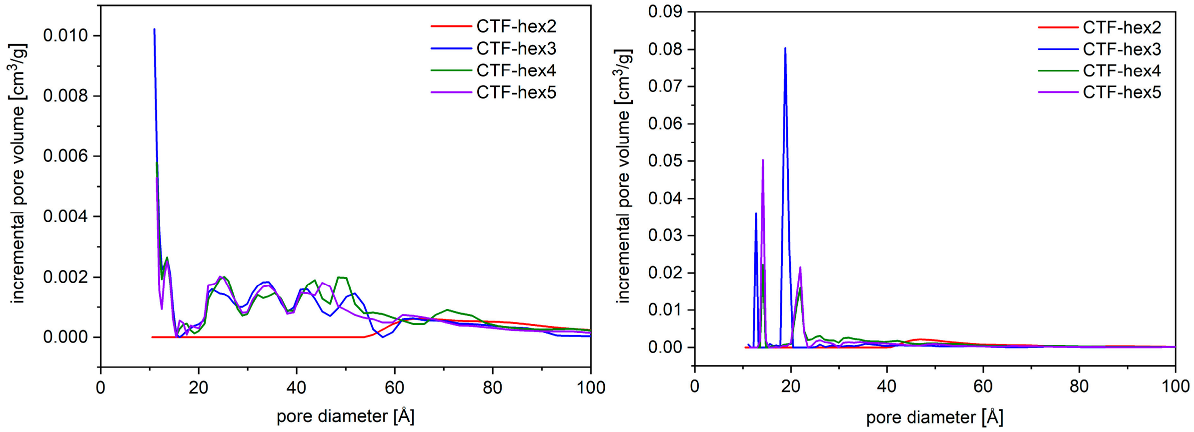

(**b**) from N_2 sorption, QSDFT, adsorption branch only ads. and des. (equilibrium model)

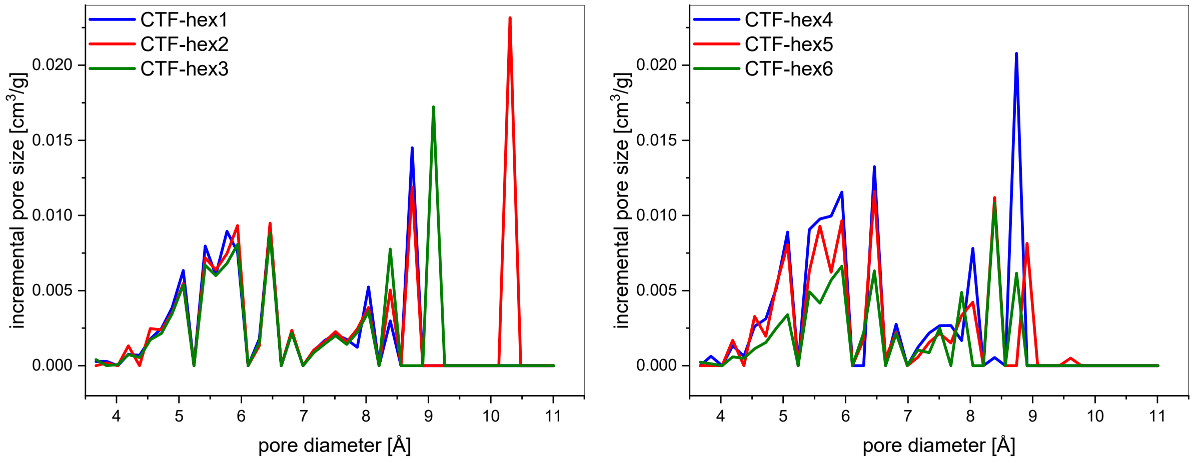

(**c**) from CO_2 adsorption

Figure S6. (**a**) NLDFT pore size distributions for **CTF-hex2–5** and **CTF-hex6** from N_2 ad- and desorption isotherms at 77 K. (**b**) QSDFT pore size distributions for **CTF-hex2–5** from N_2 adsorption isotherms at 77 K (left) and from adsorption and desorption isotherms (equilibrium model, right); note the different scale for the left and right graph. (**c**) Pore size distributions from CO_2 adsorption isotherms at 273 K. Micropores have diameters of less than 20 Å (2 nm), mesopores range from 20–500 Å (2–50 nm). The jagged lines for the micropore distribution graphs in c) must not be overinterpreted as the number of data points in the relevant low-pressure range for the CO_2 measurement was relatively low (Figure S7).

Note: for NLDFT, only an equilibrium model using the ad- and desorption branch was available, while for QSDFT besides the equilibrium model also the adsorption branch could be used only, in view of the not closed desorption isotherms (Figure S5).

4.3. CO_2 and CH_4 Adsorption-desorption Isotherms up to 1 bar

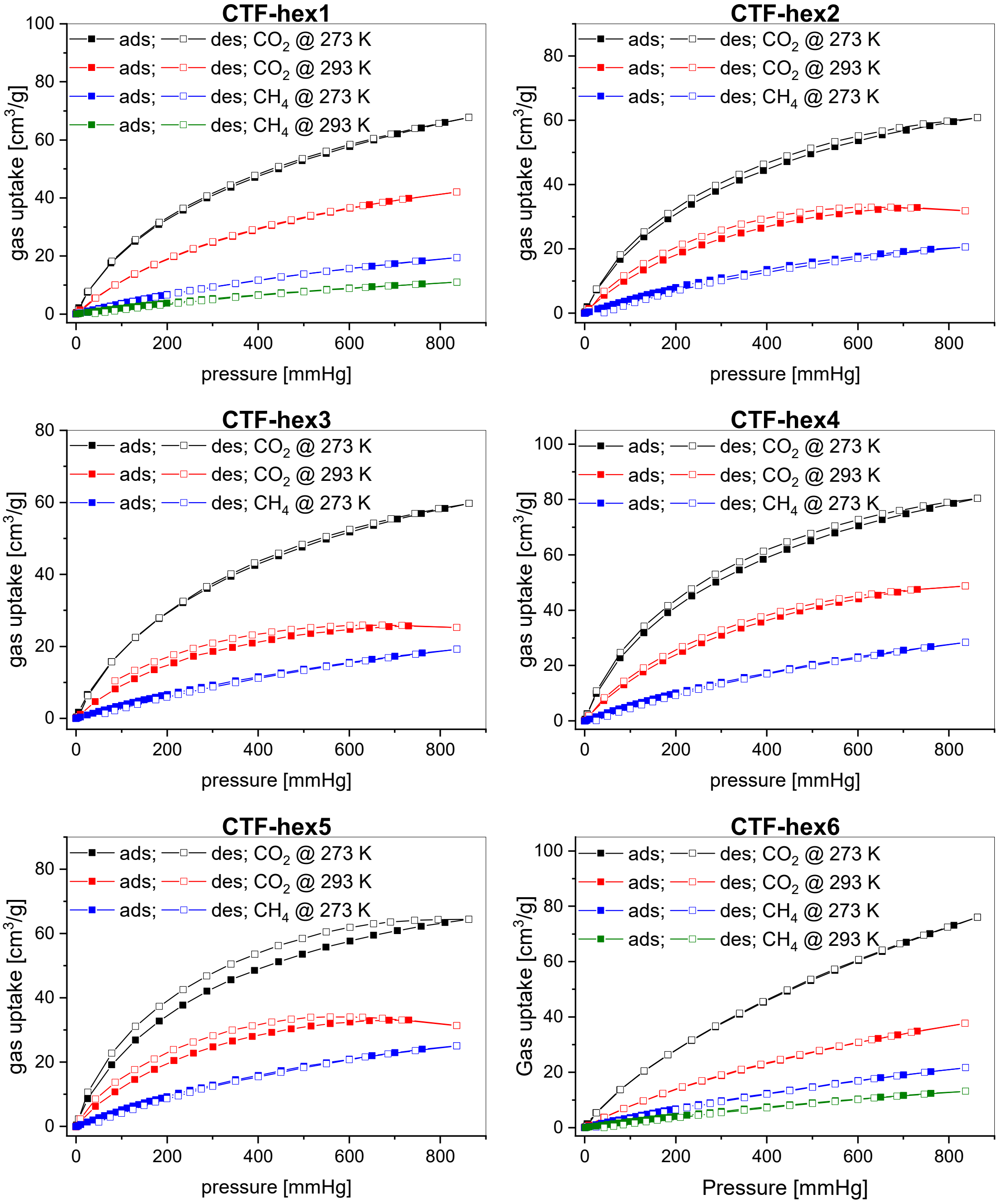

Figure S7. CO_2 and CH_4 sorption isotherms for **CTF-hex1–6** (closed symbols for adsorption and open symbols for desorption). A slightly negative slope for **CTF-hex3** and **–hex5** at 293 K is sometimes seen for materials where the gas uptake induces a phase transition.

4.4. Literature Values for CO_2 Uptake for Comparison

For comparison of the CO_2 uptake capacity, values of CO_2 adsorption properties of other CTFs are collected in Table S3.

Table S3. Surface area and CO_2 adsorption properties of triazine-based polymers.

Compound	BET [m²/g]	CO_2 uptake [mmol/g] at 1 bar		Q_{st} [a]	Ref.
		273 K	298 K or 293 K		
CTF-0	2011	4.22	-	-	[8]
CTF-1	746	2.47	1.41	27.5	[9]
CTF-1-600	1533	3.82	2.25	30.0	[9]
FCTF-1	662	4.67	3.21	35.0	[9]
FCTF-1-600	1535	5.53	3.41	32	[9]
CTF-P2	776	1.84	-	-	[7]
CTF-P3	571	2.22	-	-	[7]
CTF-P4	867	3.05	-	-	[7]
CTF-P5	960	2.94	-	-	[7]
CTF-P6	1152	3.32	-	-	[7]
CTF-P1M	4	0.92	-	-	[7]
CTF-P2M	464	1.87	-	-	[7]
CTF-P3M	523	2.21	-	-	[7]
CTF-P4M	542	1.83	-	-	[7]
CTF-P5M	542	2.03	-	-	[7]
CTF-P6M	947	4.12	-	-	[7]
MCTP-1	1452	4.61	2.65		[10]
MCTP-2	859	3.65	2.42		[10]
Polymer 2	-	-	1.56		[11]
Polymer 3	646	-	1.68		[11]
Polymer 4	1266	-	2.09		[11]
Polymer 2C	427	-	2.99		[11]
Polymer 3C	1173	-	3.08		[11]
Polymer 4C	1316	-	3.61		[11]
NOP-1	978	1.83	1.06	32.8	[12]
NOP-2	1055	2.37	1.40	34.1	[12]
NOP-3	1198	2.51	1.39	33.8	[12]
NOP-4	635	1.71	0.83	31.9	[12]
NOP-5	913	1.45	0.72	30.5	[12]
NOP-6	1130	1.31	0.49	29.2	[12]
MCTF-300	640	2.25	1.39	24.6	[13]
MCTF-400	1060	2.36	1.55	25.4	[13]
MCTF-500	1510	3.16	2.22	26.3	[13]
fl-CTF300	15	1.27	0.71	43.1	[14]
fl-CTF350	1235	4.28	2.29	32.7	[14]
fl-CTF400	2862	4.13	1.97	30.7	[14]
fl-CTF500	2322	3.26	1.65	31.7	[14]
fl-CTF600	2113	3.48	1.80	32.4	[14]
COP-1	168	-	1.33	-	[15]
COP-2	158	-	0.91	-	[15]
PCTF-1	2235	3.21	1.84	30	[16,17]
PCTF-2	784	1.83	0.992	26	[16,17]
PCTF-3	641	2.14	1.32	27	[16,17]
PCTF-4	1090	2.27	1.49	28	[16,17]

PCTF-5	1183	2.55	1.48	27	[16,17]
PCTF-6	79	-	-	-	[16,17]
PCTF-7	613	2.15	1.32	25	[16,17]
HPF-1	576	-	2.8	43	[18]
CTF-TPC	1668	4.19	2.47	32	[19]
CTF-FL	773	3.22	2.00	35	[19]
CTF-FUM-350	230	3.44	2.31	-	[20]
CTF-DCN-500	735	2.66	1.55	-	[20]
pym-CTF500	208	2.75	1.77	40.5	[21]
pym-CTF600	689	3.34	2.15	37.4	[21]
bipy-CTF300-600	360-2479	1.87-5.58	0.98-2.95	33.3-35.2	[21]
lut-CTF300-600	486-2815	3.63-4.99	2.14-2.52	33.3-38.2	[21]
Ad4L1	1617	3.36	1.97	34.3	[22]
Ad4L2	1885	2.47	1.94	32.6	[22]
Ad4L3	1341	3.28	1.42	37.1	[22]

[a] heat of adsorption (negative adsorption enthalpy).

5. Calculation of Isosteric Heat of Adsorption (Q_{st}) using Virial Fitting Method

The isosteric heat of adsorption (Q_{st}) for the CO_2 adsorption of **CTF-hex1–6** were calculated from isotherms at different temperatures (273 K and 293 K), applying the virial fitting method (Equation (1)).

$$\ln P = \ln N + \frac{1}{T}\sum_{i=0}^{m} a_i N^i + \sum_{i=0}^{n} b_i N^i \quad (1)$$

In Equation (1), P is the pressure expressed in Torr, N is the amount adsorbed in mmol/g, T is the temperature in K, a_i and b_i are virial coefficients, and m, n represents the number of coefficients required to describe the isotherms adequately. Then values of the virial coefficients a_0 through a_m were employed to calculate the Q_{st} using the following expression (Equation (2)).

$$Q_{st} = -R\sum_{i=0}^{m} a_i\, N^i \quad (2)$$

R is the universal gas constant with R = 8.314 J/(K mol).

The Q_{st} at zero coverage (Q^0_{st}) is calculated according to (Equation (3)).

$$Q_{st} = -R\sum_{i=0}^{m} a_0 \quad (3)$$

Table S3. List of a_0 and Q^0_{st} values of the CO_2 adsorption isotherms for **CTF-hex1-6** obtained from the virial fitting method.

Entry	CTF-hex	a_0	Q^0_{st} (CO_2) [kJ/mol]
1	CTF-hex1	−3964.67286	33
2	CTF-hex2	−2770.73334	23
3	CTF-hex3	−3857.17197	32
4	CTF-hex4	−3475.35261	29
5	CTF-hex5	−3534.26034	29
6	CTF-hex6	−4450.11578	37

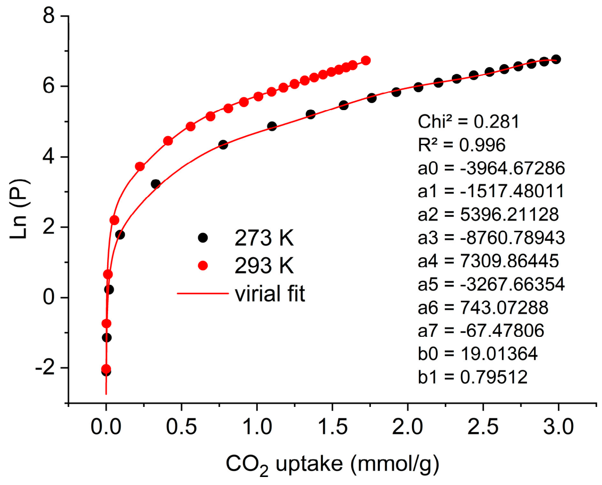

Figure S8. CO_2 adsorption isotherms for **CTF-hex1** fitted by the virial method.

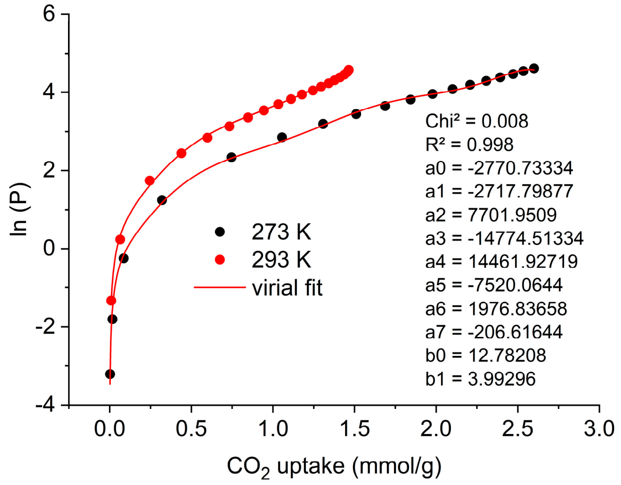

Figure S9. CO_2 adsorption isotherms for **CTF-hex2** fitted by the virial method.

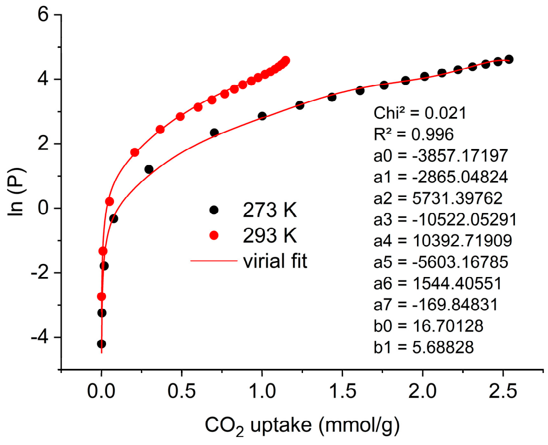

Figure S10. CO_2 adsorption isotherms for **CTF-hex3** fitted by the virial method.

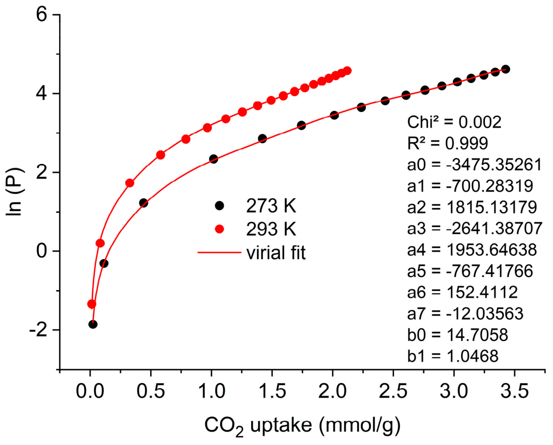

Figure S11. CO_2 adsorption isotherms for **CTF-hex4** fitted by the virial method.

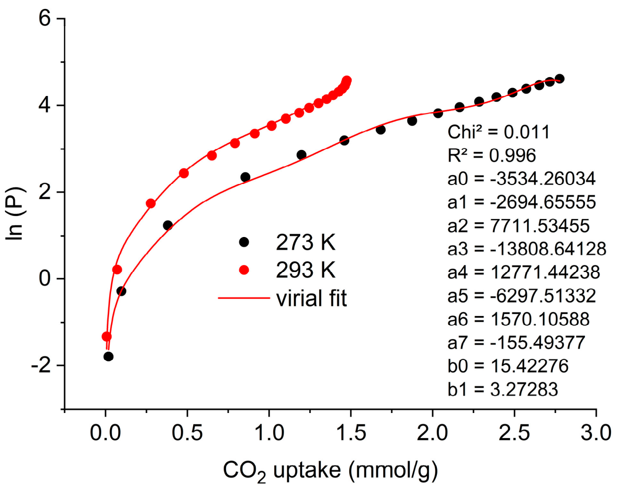

Figure S12. CO_2 adsorption isotherms for **CTF-hex5** fitted by the virial method.

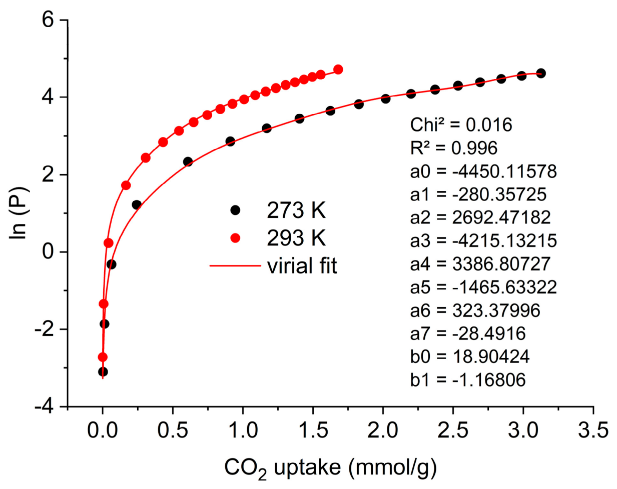

Figure S13. CO_2 adsorption isotherms for **CTF-hex6** fitted by the virial method.

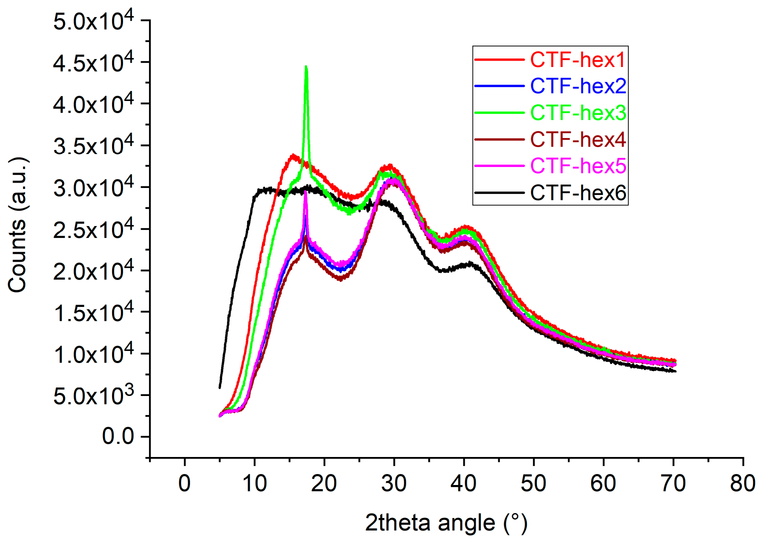

Figure S14. Powder X-ray diffractograms of **CTF-hex1–6**. In other work on mixed-linker CTFs which were prepared by ionothermal synthesis with $ZnCl_2$,[22][23] the comparatively sharp peak at 2θ = 17° was assigned to the (111) plane reflection from $ZnCl_2$. This assignment was not correct, however, in view of the synthesis of mixed-linker **CTF-hex2–5** with the Brønsted acid trifluoro-methanesulfonic acid. At present, the origin of this sharp reflection at 2θ = 17° remains unclear.

References

1. Clark, W.; Still, W.C.; Kahn, M.; Mitra, A. Rapid chromatographic technique for preparative separations with moderate resolution. *J. Org. Chem.* **1978**, *43*, 2923–2925.
2. Plietzsch, O.; Schilling, C. .; Tolev, M.; Nieger, M.; Richert, C.; Muller, T.; Bräse, S. Four-fold click reactions: Generation of tetrahedral methane- and adamantane-based building blocks for higher-order molecular assemblies. *Org. Biomol. Chem.* **2009**, *7*, 4734–4743.
3. Plietzsch, O.; Schade, A.; Hafner, A.; Huuskonen, J.; Rissanen, K.; Nieger, M.; Muller, T.; Bräse, S. Synthesis and Topological Determination of Hexakis-Substituted 1,4-Ditritylbenzene and Nonakis-Substituted 1,3,5-Tritritylbenzene Derivatives: Building Blocks for Higher Supramolecular Assemblies. *Eur. J. Org. Chem.* **2013**, 283–299, doi:10.1002/ejoc.201201162.
4. Li, L.-H.; Pan, Z.-L.; Duan, X.-H.; Liang, Y.-M. An Environmentally Benign Procedure for the Synthesis of Aryl and Arylvinyl Nitriles Assisted by Microwave in Ionic Liquid. *Synlett* **2006**, *2006*, 2094–2098.
5. Becker, M.; Schulz, A.; Voss, K. Synthesis of poly-nitrile aromatics via palladium-catalyzed cyanation of aryl bromides with potassium hexacyanoferrate(II). *Synth. Commun.* **2011**, *41*, 1042–1051.
6. Plietzsch, O. *Modulare Synthesen Funtionalisierter Tetraphenylmethanderivate als Bausteine für Supramolekulare Netzwerke*; Logos Verlag: Berlin, Germany, 2010. https://publikationen.bibliothek.kit.edu/1000021769
7. Ren, S.; Bojdys, M.J.; Dawson, R.; Laybourn, A.; Khimyak, Y.Z.; Adams, D.J.; Cooper, A.I. Porous, fluorescent, covalent triazine-based frameworks via room-temperature and microwave-assisted synthesis. *Adv. Mater.* **2012**, *24*, 2357–2361.
8. Katekomol, P.; Roeser, J.; Bojdys, M.; Weber, J.; Thomas, A. Covalent Triazine Frameworks Prepared from 1,3,5-Tricyanobenzene. *Chem. Mater.* **2013**, *25*, 1542–1548.
9. Zhao, Y.; Yao, K.X.; Teng, B.; Zhang, T.; Han, Y. A perfluorinated covalent triazine-based framework for highly selective and water–tolerant CO_2 capture. *Energy Environ. Sci.* **2013**, *6*, 3684.
10. Puthiaraj, P.; Cho, S.-M.; Lee, Y.-R.; Ahn, W.-S. Microporous covalent triazine polymers: Efficient Friedel-Crafts synthesis and adsorption/storage of CO_2 and CH_4. *J. Mater. Chem. A* **2015**, *3*, 6792–6797.
11. Lim, H.; Cha, M.C.; Chang, J.Y. Preparation of Microporous Polymers Based on 1,3,5-Triazine Units Showing High CO_2 Adsorption Capacity. *Macromol. Chem. Phys.* **2012**, *213*, 1385–1390.
12. Xiong, S.; Fu, X.; Xiang, L.; Yu, G.; Guan, J.; Wang, Z.; Du, Y.; Xiong, X.; Pan, C. Liquid acid-catalysed fabrication of nanoporous 1,3,5-triazine frameworks with efficient and selective CO_2 uptake. *Polym. Chem.* **2014**, *5*, 3424.
13. Liu, X.; Li, H.; Zhang, Y.; Xu, B.; Sigen, A.; Xia, H.; Mu, Y. Enhanced carbon dioxide uptake by metalloporphyrin-based microporous covalent triazine framework. *Polym. Chem.* **2013**, *4*, 2445.
14. Hug, S.; Mesch, M.B.; Oh, H.; Popp, N.; Hirscher, M.; Senker, J.; Lotsch, B.V. A fluorene based covalent triazine framework with high CO_2 and H_2 capture and storage capacities. *J. Mater. Chem. A* **2014**, *2*, 5928–5936.
15. Patel, H.A.; Karadas, F.; Canlier, A.; Park, J.; Deniz, E.; Jung, Y.; Atilhan, M.; Yavuz, C.T. High capacity carbon dioxide adsorption by inexpensive covalent organic polymers. *J. Mater. Chem.* **2012**, *22*, 8431.
16. Bhunia, A.; Boldog, I.; Möller, A.; Janiak, C. Highly stable nanoporous covalent triazine-based frameworks with an adamantane core for carbon dioxide sorption and separation. *J. Mater. Chem. A* **2013**, *1*, 14990.

17. Bhunia, A.; Vasylyeva, V.; Janiak, C. From a supramolecular tetranitrile to a porous covalent triazine-based framework with high gas uptake capacities. *Chem. Commun.* **2013**, *49*, 3961.
18. Nandi, S.; Werner-Zwanziger, U.; Vaidhyanathan, R. A triazine-resorcinol based porous polymer with polar pores and exceptional surface hydrophobicity showing CO_2 uptake under humid conditions. *J. Mater. Chem. A* **2015**, *3*, 21116–21122.
19. Bhunia, A.; Esquivel, D.; Dey, S.; Fernández-Terán, R.; Goto, Y.; Inagaki, S.; Van Der Voort, P.; Janiak, C. A photoluminescent covalent triazine framework: CO_2 adsorption, light-driven hydrogen evolution and sensing of nitroaromatics. *J. Mater. Chem. A* **2016**, *4*, 13450–13457.
20. Wang, K.; Huang, H.; Liu, D.; Wang, C.; Li, J.; Zhong, C. Covalent Triazine-Based Frameworks with Ultramicropores and High Nitrogen Contents for Highly Selective CO_2 Capture. *Environ. Sci. Technol.* **2016**, *50*, 4869–4876.
21. Hug, S.; Stegbauer, L.; Oh, H.; Hirscher, M.; Lotsch, B.V. Nitrogen-Rich Covalent Triazine Frameworks as High-Performance Platforms for Selective Carbon Capture and Storage. *Chem. Mater.* **2015**, *27*, 8001–8010.
22. Dey, S.; Bhunia, A.; Boldog, I.; Janiak, C. A mixed-linker approach towards improving covalent triazine-based frameworks for CO_2 capture and separation. *Microporous Mesoporous Mater.* **2017**, *241*, 303–315.
23. Dey, S.; Bhunia, A.; Breitzke, H.; Groszewicz, P.B.; Buntkowsky, G.; Janiak, C. Two linkers are better than one: Enhancing CO_2 capture and separation with porous covalent triazine-based frameworks from mixed nitrile linkers. *J. Mater. Chem. A* **2017**, *5*, 3609–3620.
